# Transcriptomic comparison between two *Vitis vinifera* L. varieties (Trincadeira and Touriga Nacional) in abiotic stress conditions

**DOI:** 10.1186/s12870-016-0911-4

**Published:** 2016-10-12

**Authors:** Margarida Rocheta, João L. Coito, Miguel J. N. Ramos, Luísa Carvalho, Jörg D. Becker, Pablo Carbonell-Bejerano, Sara Amâncio

**Affiliations:** 1 Instituto Superior de Agronomia, LEAF, Linking Landscape, Environment, Agriculture and Food, Universidade de Lisboa, 1349-017 Lisboa, Portugal; 2 Instituto Gulbenkian de Ciência, 2780-156 Oeiras, Portugal; 3 Instituto de Ciencias de la Vid y del Vino, CSIC-Universidad de La Rioja-Gobierno de la Rioja, 26007 Logroño, Spain

**Keywords:** Grapevine, Field Conditions, Controlled Conditions, Microarray, Gene expression, Varietal diversity

## Abstract

**Background:**

Predicted climate changes announce an increase of extreme environmental conditions including drought and excessive heat and light in classical viticultural regions. Thus, understanding how grapevine responds to these conditions and how different genotypes can adapt, is crucial for informed decisions on accurate viticultural actions. Global transcriptome analyses are useful for this purpose as the response to these abiotic stresses involves the interplay of complex and diverse cascades of physiological, cellular and molecular events. The main goal of the present work was to evaluate the response to diverse imposed abiotic stresses at the transcriptome level and to compare the response of two grapevine varieties with contrasting physiological trends, Trincadeira (TR) and Touriga Nacional (TN).

**Results:**

Leaf transcriptomic response upon heat, high light and drought treatments in growth room controlled conditions, as well as full irrigation and non-irrigation treatments in the field, was compared in TR and TN using GrapeGene GeneChips®. Breakdown of metabolism in response to all treatments was evidenced by the functional annotation of down-regulated genes. However, *circa* 30 % of the detected stress-responsive genes are still annotated as «Unknown» function. Selected differentially expressed genes from the GrapeGene GeneChip® were analysed by RT-qPCR in leaves of growth room plants under the combination of individual stresses and of field plants, in both varieties. The transcriptomic results correlated better with those obtained after each individual stress than with the results of plants from field conditions.

**Conclusions:**

From the transcriptomic comparison between the two Portuguese grapevine varieties Trincadeira and Touriga Nacional under abiotic stress main conclusions can be drawn: 1. A different level of tolerance to stress is evidenced by a lower transcriptome reprogramming in TN than in TR. Interestingly, this lack of response in TN associates with its higher adaptation to extreme conditions including environmental conditions in a changing climate; 2. A complex interplay between stress transcriptional cascades is evidenced by antagonistic and, in lower frequency, synergistic effects on gene expression when several stresses are imposed together; 3. The grapevine responses to stress under controlled conditions are not fully extrapolated to the complex vineyard scenario and should be cautiously considered for agronomic management decision purposes.

**Electronic supplementary material:**

The online version of this article (doi:10.1186/s12870-016-0911-4) contains supplementary material, which is available to authorized users.

## Background

Grapevine (*Vitis vinifera* L.) is among the most economically important crops worldwide. According to the International Organization of Vine and Wine, in 2013, grapevine occupied more than 7,500 thousand hectares in cultivated areas. Nevertheless, in 2012, wine production decreased by 6 % in Europe, mainly due to weather conditions (http://www.oiv.int/en/). Although Mediterranean regions offer among the best climate conditions for viticulture [1, 2], the soil and atmospheric water deficits along with high summer temperatures can have a negative impact on crop yield and fruit quality [3]. Furthermore, it is expected that impending climate changes may significantly impair grapevine production and quality [1, 4, 5].

The major and most relevant abiotic stresses that can affect grapevine production in the Mediterranean region are drought, excessive light and excessive heat [6]. In field conditions it is rare that plants are affected by only one abiotic stress. Plants subjected to drought are usually also affected by heat and, sometimes, by excessive light which can cause photoinhibition [7]. Plants, as sessile organisms, are able to set in motion several mechanisms to deal with and to overcome environmental constraints. Response to abiotic stress is highly complex and involves the interplay of different responses at plant and cell levels. A cascade of molecular, cellular and physiological events can occur simultaneously and very rapidly. However, experimental approaches show that the processes triggered by each individual abiotic stress differ significantly and show little overlap [7, 8]. In recent years, many advances have been made towards understanding how plants respond to abiotic stresses, individually or in combination [9, 10]. Although large amounts of data on the expression of genes related to abiotic stress are available, the challenge now is to connect those genetic profiles to changes in plant physiology. Concerning grapevines, the varieties Touriga Nacional (TN) and Trincadeira (TR) are known to be among the most important native varieties in Portugal, used to produce high quality red wines. Trincadeira is widely cultivated in the south of Portugal as it grows well in hot, dry and bright areas while TN, formerly cultivated in the north of Portugal [11] is nowadays cultivated throughout the whole Portuguese territory. The choice of these varieties was brought about due to contrasting physiological responses to stress. Touriga Nacional has a higher capacity to dissipate heat through evaporative cooling and is better adapted to warm climate conditions, as long as no water stress occurs [12]. Upon heat stress, TR is more intensely affected and for a longer period than TN, up-regulating several anti-oxidative stress genes [13]. In addition, a high throughput search for transcriptomic responses increases the chance of finding key regulatory genes and proteins [7]. Usually the first processes to be affected by abiotic stress are photosynthesis and cell growth with subsequent issues in plant development. These effects can be either direct, by a decrease in available CO_2_ due to stomata closure, or indirect, by the onset of oxidative stress, a secondary effect of most abiotic stresses, which can be deleterious to the photosynthetic machinery and to other cellular mechanisms [7]. All these responses are also described as contributing to acclimation, and then to the alleviation of abiotic stress damage [9, 10, 14, 15]. Recent advances in understanding the response to abiotic stress have unravelled several cell signalling pathways interconnected at many levels. They were clearly revealed by approaches using the combination of abiotic stresses [9, 14] which affect the expression of hundreds of genes [8, 16, 17]. Considering transcriptomic microarray projects in grapevines under abiotic stresses it is possible to quote reports of studies conducted with leaves focusing on heat, cold, drought or excessive light [8, 18–20]. Meanwhile a comparative analysis of grapevine gene prediction introduced substantial progress in *Vitis* genome annotation and provided a significant incentive for novel transcriptomic studies [21]. In the present study, a transcriptomic analysis was performed on leaves of TR and TN in order to compare their response at gene expression level 1) upon the application of individual abiotic stress treatments (drought, W; heat, H; high light, L) in growth room controlled conditions and 2) upon full irrigation (FI) versus no irrigation (NI) in hot and dry summer field conditions to test for the first time with these varieties, how irrigation can change transcriptomic response. To complement the microarray analysis of the three abiotic stresses, the expression of the most highly up- or down-regulated genes pinpointed through the array was quantified by RT-qPCR in leaves of growth room plants subjected to the combination of the abiotic stresses in pairs or in triplets. The rationale of this experiment was that individual stresses interact with each other after combined application, so the transcription of the set of genes that respond to controlled individual, combined or field imposed abiotic stress, was compared in TR and TN grapevine varieties.

## Results and discussion

### Trincadeira and Touriga Nacional show distinct physiological responses to abiotic stress

**Fig. 1:**
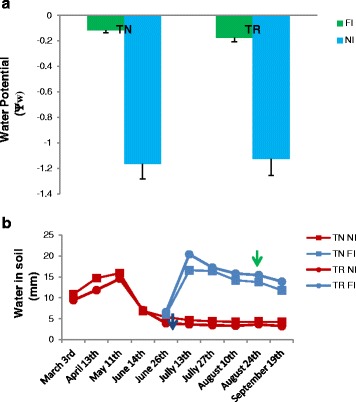
Leaf water potential of field plants and soil water content. Field water potential (**a**, Ψw) in irrigated and non-irrigated plants of Trincadeira and Touriga Nacional before leaf collection. Water present in the soil (**b**, mm) over duration of the field trial. Blue arrow indicates the time when irrigated plants (FI) started to receive water and green arrow indicates the day when leaves were collected. FI, Full irrigation; NI, Non irrigation. TR, Trincadeira; TN, Touriga Nacional

In our study, the first approach was to ascertain whether the stress treatments had in fact induced a physiological response. Chlorophyll fluorescence parameters reflect the maximum efficiency of PSII photochemistry in dark- and in light-adapted leaves (respectively, Fv/Fm and F’v/F’m). In growth room experiments, these parameters were affected by stress, suffering significant decreases in both varieties Trincadeira (TR) and Touriga Nacional (TN), although in a unique pattern in each variety (Table 1). Touriga Nacional was more significantly affected by individual stresses than TR while in this variety only double or triple stresses caused significant decreases. Similar in both varieties was the effect of heat stress (H) as individual or combined treatment that indicate a direct influence on the photosynthetic apparatus (Table 1), similar to photosynthesis alteration in situations when drought was combined with other stresses, especially in TR [13, 22]. In field plants, pre-dawn leaf water potential and soil water content, confirmed the severe water stress affecting non irrigated plants in both varieties (Fig. 1a and b; Additional file 1). Previous results report that TN can withstand growth in warmer climates with higher levels of irradiance than TR, as long as water is available [12, 23]. In these conditions TN maintains higher photosynthesis rates and chlorophyll fluorescence parameters than TR, which point to the absence of severe stress in TN [23].

**Table 1:** Chlorophyll fluorescence parameters measured in the two grapevine varieties, Touriga Nacional (TN) and Trincadeira (TR)

	Fv/Fm					F’v/F’m				
	TN	TR	a	b	c	TN	TR	a	b	c
Control	0.78	0.79	-	-	^a^	0.65	0.67	-	-	
	0.01	0.01				0.02	0.03			
W	0.62	0.69				0.59	0.61	^a^		
	0.16	0.09				0.02	0.03			
L	0.62	0.56	^a^			0.49	0.56	^a^		
	0.12	0.16				0.09	0.12			
H	0.53	0.52	^a^			0.52	0.47		^a^	
	0.14	0.16				0.10	0.14			
WL	0.45	0.62	^a^			0.38	0.51	^a^	^a^	
	0.15	0.11				0.14	0.07			
WH	0.52	0.55	^a^	^a^		0.50	0.48		^a^	
	0.15	0.13				0.11	0.11			
LH	0.57	0.62	^a^	^a^		0.51	0.63	^a^		^a^
	0.13	0.12				0.08	0.02			
WLH	0.57	0.59	^a^	^a^		0.48	0.48	^a^	^a^	
	0.13	0.12				0.08	0.08			

Plants were subjected to individual (water, W; light, L; heat, H) and combined stresses (WL; WH; LH; WLH) as indicated in Material and Methods. *F*_*v*_ */F*_*m*_ represents the maximum efficiency of PSII photochemistry in darkadapted leaves and *F’*_*v*_ */F’*_*m*_ corresponds to the maximum quantum efficiency of PSII in light-adapted leaves. Values are accompanied by the respective standard errors. Statistically significant differences after Tukey’s multiple comparison tests for *p <* 0.05 are the following: ^**a**^ in column a: significant difference between TN and the respective control; ^**a**^ in column b: significant difference between TR and the respective control; ^**a**^ in column c: significant difference between TN and TR within a stress treatment

### PCA and HCA analysis of microarray gene expression data show differential stress response tendencies

Transcriptome profiling was carried out in Trincadeira (TR) and Touriga Nacional (TN) leaf samples from the six different conditions tested: control, C; water deficit, W; heat, H; high light, L in the growth room, as well as upon full irrigation (FI) and no irrigation (NI) in the field vineyard. Three replicates per sample type as in Material and Methods were analyzed using GrapeGen Genechip microarrays, which represent more than 17 k grapevine unigenes [24].

**Fig. 2:**
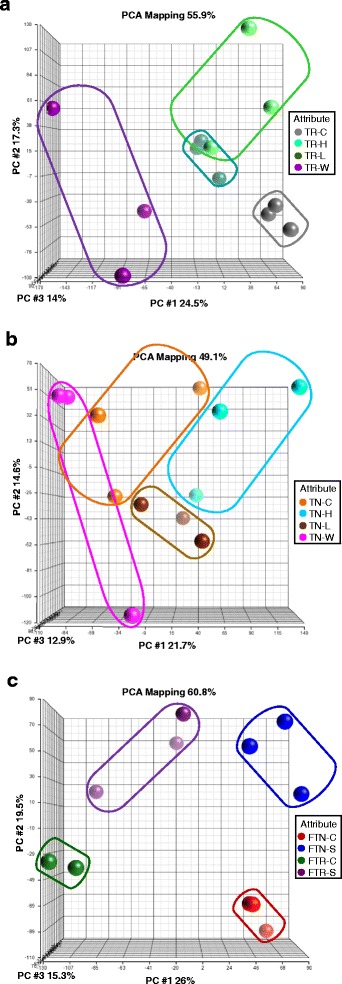
Principal Component Analysis (PCA) of microarray results in growth room and field experiments. The PCA were performed with normalized expression of all the transcripts. **a** Trincadeira (TR) growth room; **b** Touriga Nacional (TN) growth room; **c** Field experiment with both varieties; −C, growth room control; −H, growth room heat stress; −L, growth room high light radiation stress; −W, growth room water deficit. FTN-C, Field full irrigation Touriga Nacional, control; FTN-S, Field non-irrigation Touriga Nacional, stress; FTR-C, Field full irrigation Trincadeira, control; FTR-S, Field non-irrigation Trincadeira, stress

The microarray data was first analysed by means of principal component analysis (PCA) (Fig. 2) and hierarchical clustering analysis (HCA) (Additional file 2) in order to access the similarity of the sample replicates for each treatment and to identify the main sources of gene expression variation [25]. From the PCA of TR samples, the plot of principal component (PC) 1 (24.5 % of the variability) depicts a marked difference between water deficit (W) and the other conditions. Furthermore, a slight separation between control and L stress samples as well as two H stress replicates was observed, so it might be assumed that the strength of the stress response is explained by PC1 in Trincadeira individual stress (IS) samples. Also for Trincadeira IS samples, PC2 (17.3 % of the variability) depicts variability within W and H replicates (Fig. 2a). Similarly, hierarchical cluster analysis (HCA) showed a separation between W and all other growth room TR samples. Moreover, HCA showed consistency between replicates in all conditions assayed for TR (Additional file 2). In Touriga Nacional IS samples a high variation was observed within replicates after all treatments except L (Fig. 2b), PC1 explaining 21.7 % and PC2 14.6 % of the differences. The lack of homogeneity in TN samples can be assigned to a less clear effect of the treatments. This observation is further supported by the HCA (Additional file 2) where the replicates from different conditions did not cluster together. Regarding the PCA of field samples, PC1 (26 % of variation) separated the varieties and PC2 (19.5 %) the irrigation regimes (Fig. 2c). Noticeably, lower response of TR to the field water deprivation treatment is evident in the separation of the samples in PC2. The three replicates of each variety cluster together upon the different experimental conditions, a pattern confirmed in the HCA (Additional file 2). In fact, the HCA showed consistency between replicates of both TN and TR field conditions that in turn were clearly separated from all growth room samples irrespective- of the genotype. The results confirm the different behaviour between TN and TR, which could be explained by the basal tolerance of TN [12, 13].

### Differential gene expression response between controlled and field stress conditions

**Fig. 3:**
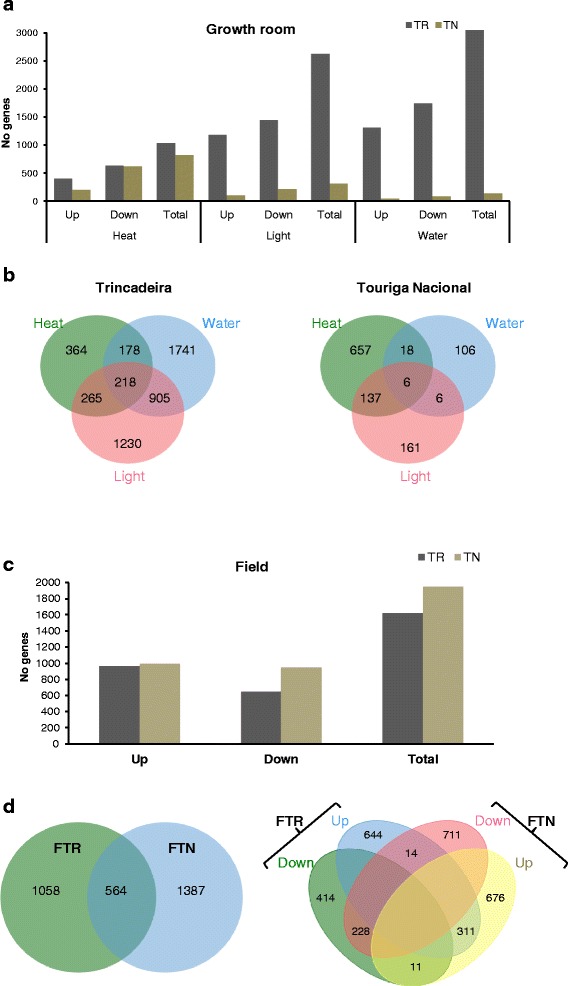
Differentially expressed genes in growth room and in field experiments. **a** Number of total, up- or down-regulated genes of the microarray showing significant expression changes after individual stresses in comparison to the control. **b** Venn diagrams showing the number of genes distinct or common to the different individual stresses. **c** Number of total, up- or down-regulated genes of the microarray showing significant expression changes in the field trial. **d** Venn diagrams showing the number of transcripts distinct or common to both varieties (left) and the up or down-regulated transcripts distinct or common to both varieties in the field stress trial in relation to control (right). Heat, High light, Water deficit in Trincadeira (left) and Touriga Nacional (right). Top, up-regulated genes; Bottom, down-regulated genes; TR, Trincadeira; TN, Touriga Nacional; FTN, Field Touriga Nacional; FTR, Field Trincadeira

Transcripts significantly changing expression in response to water deficit (W), heat (H) and high light (L) stress under controlled conditions, as well as field no irrigation (NI) treatment, were searched by comparison to the respective control samples (5 % False Discovery Rate - FDR). Remarkably, more down- than up-regulated genes were generally detected in response to individual stresses (ISs) in both varieties (Fig. 3a). However the number of responsive genes was significantly lower in TN in particular after W and L stress, respectively 136 and 318 in TN versus 3042 and 2618 in TR (Fig. 3b). High light and H are the stresses that showed the highest number of gene expression responses shared between the two varieties, 31 in total (most of them down-regulated) (Additional file 3). These shared responsive genes code for an ELIP, a heat shock protein (HSP), an ethylene responsive factor (ERF), a nudix hydrolase and a calcium binding protein. It is interesting to highlight the transcripts coding for one zinc finger containing protein, one nudix hidrolase (*NUDT17*) and two ß-expansin annotated as *VviEXPA18* and *VviEXLB4* [26] Zinc finger proteins belong to a large eukaryotic TF family sharing CnHn motifs, which are involved in plant growth and development and also in responses to environmental stresses [27]. Three transcripts down-regulated by H and L, in both varieties, share the C3HC4 type Zn-finger (RING-finger) domain. C3HC4 is one of the TF sub-families up-regulated in response to light stress and has been defined as a specific ROS marker [28]. However, studies in grapevine have already shown an opposite response (down-regulation under drought and heat [8], and under high light [18]). The present work further confirms these findings in both studied varieties. Nudix hydrolases are ubiquitous enzymes that hydrolyse a large variety of nucleoside diphosphate derivatives [29, 30]. The protein NUDT17 has been associated with biotic stress in *Arabidopsis thaliana* [31] and recently, a cytoplasm RhNudix1was found to catalyse a step in the pathway for scent monoterpenesin in roses [32]. Cell wall genes, namely those coding for expansins, pectinesterases, and endoxyloglucan transferases are usually down-regulated in typical abiotic stress responses when cell division and growth are hindered [33], and thus it is not surprising to find a ß-expansin among the genes down-regulated in both varieties and in two different abiotic stresses (L and H).

The varieties reacted differently in the field experiment. In fact, conversely to IS and in agreement with the PCA plot, the total number of differentially expressed genes was slightly higher in TN (1951) than in TR (1622) field plants (Fig. 3c), 311 up-regulated and 228 down-regulated under NI in both varieties (Fig. 3d). The results obtained indicate that, under the same field conditions each variety expressed specific sets of genes, whereas only a minor proportion of the stress response was shared. However, when transcripts responsive to the field NI treatment were directly compared to the IS responsive ones, the results evidenced a variety-dependent response (Additional file 4). In Trincadeira, although the number of W and L-responsive genes was higher than in NI, only a small proportion of IS and NI-responsive genes overlapped (Additional file 4a–c). Focusing on TN plants we observe a distinct behaviour: a lower number of IS-responsive genes but a higher proportion overlapping with the NI responsive ones (Additional file 4d–f). A species-associated response was recently described for desiccated leaves of three *Vitis* species, which differed in activation of ABA and ethylene signalling pathways according to their sensitivity or tolerance to drought [34]. A variety-associated response has been previously described for metabolite accumulation in Cabernet Sauvignon and Sangiovese berries [35]. Furthermore a transcriptomic varietal specificity comprising 180 novel genes not found in the already sequenced grapevine varieties was identified in the Italian cv. Corvina [36].

### Distribution of differentially expressed genes into functional categories is similar in both varieties

Treatment-responsive transcripts were assigned into seven functional categories (Cellular process, Metabolism, Regulation, Response to stimulus, Signalling, Transport, and Unknown) according to GrapeGen 12x_v2.1 annotation (http://genomes.cribi.unipd.it/grape/) (Additional file 5). In Trincadeira (TR) under individual stress (IS), most functional categories presented more down- than up-regulated genes. Exceptionally, Response to stimulus and Regulation (in W) and Signalling and Transport (in L) showed more up-regulated genes. Additionally, in H most genes were down-regulated in all functional categories (Additional file 5). These results differ from a previous study with the grapevine variety Aragonez (syn Tempranillo) in equivalent IS conditions, where H offered a higher amount of responsive genes as compared to W [8]. With few exceptions, each stress treatment caused a higher number of down-regulated genes in all functional categories, which differs significantly from other studies in which up-regulated genes were prevalent [8, 37]. Metabolism was the category including the highest number of down-regulated genes (Additional file 5), consequently metabolism breakdown dominated plant responses.

Despite the fact that TN presented fewer responsive genes in controlled IS conditions when compared with TR (Fig. 3B), the percentage of genes in three relevant functional categories, Response to Stimulus (stress response), Signalling (hormone and other signalling pathways) and Regulation (transcription factors) was compared between the two varieties (Additional file 6). The functional annotation of up-regulated genes shows that water stress (W) gave the highest percentage in Regulation category in TN and in Signalling category in TR, a contrasting response to the IS treatments between the two varieties. Conversely, Trincadeira under high light (L) and heat (H) showed a higher percentage of up-regulated genes in Regulation. However, both varieties had similar percentages of down-regulated genes upon all stresses (Additional file 6). As a whole, different quantitative and qualitative responses to the IS treatments were observed between the two varieties. Trincadeira activated a greater transcriptome reprogramming than TN to cope with the same environmental conditions, namely investing more in enzymes and metabolites of the antioxidative system as reported for the same varieties under heat stress [13]. The smallest reprogramming in TN is enough to react more rapidly and efficiently than TN, confirming the better adaptability of this variety as before [12]. However, when comparing TN and TR under field NI conditions, the percentage of responsive genes annotated in the selected categories tended to be higher than for IS in both varieties, with the exception of down-regulated genes after H stress (Additional file 6). These results evidence the contrasting responses of both varieties between controlled and field conditions.

### Stress-responsive genes showing the greatest magnitude of change are variety-specific

For each variety, *circa* 30 genes most up- (Table 2) or down-regulated (Table 3), based on the expression fold change (treatment/control) were selected from the microarray. Ten were assigned to each individual stress (IS) treatment (five up and five down-regulated). This selection included genes without annotation (Unknown category in GrapegenDB) when homologous transcripts were identified at NCBI database (http://www.ncbi.nlm.nih.gov/) (Tables 2 and 3; Additional file 7).

**Table 2:** Up-regulated genes

Variety/Treatment	Name	Log_2_	ID	Annotation
TR_W	*VviEXLB4*	7.24	VIT_200s1455g00010	expansin-like b1
TR_W	*lEaP* ^a^	5.02	VIT_205s0020g00840	late embryogenesis abundant protein d-29-like
TR_W	*VviMYBC2-L4*	4.02	VIT_217s0000g02650	transcription repressor MYB4-like
TR_W	***HSP20***	4.39	VIT_209s0002g06790	hsp20-like chaperone
TR_W	*FMT*	4.21	VIT_210s0003g00480	flavonoid o-methyltransferase related
TR_L	*OST1*	3.63	VIT_202s0236g00130	serine threonine-protein kinase
TR_L	*ProOx* ^a^	3.55	VIT_214s0083g00520	proline dehydrogenase
TR_L	*CXE* ^a^	3.52	VIT_208s0032g00700	probable carboxylesterase 15
TR_L	*ZFC*	3.19	VIT_216s0098g00360	zinc finger protein constans-like protein
TR_L	**ELIP1**	2.96	VIT_205s0020g04110	early light-inducible protein
TR_H	*HSP21*	5.56	VIT_216s0098g01060	chloroplast low molecular weight heat shock protein
TR_H	*mHSP23*	4.73	VIT_202s0154g00490	mitochondrial small heat shock protein
TR_H	*GolS1*	4.64	VIT_207s0005g01980	galactinol synthase
TR_H	*HSP18*	4.47	VIT_213s0019g03000	18.1 kDa class i heat shock protein HSP18
TR_H	***HSP20***	4.39	VIT_209s0002g06790	hsp20-like chaperone
FTR	*PPP* ^a^	4.90	XM_010667183	pentatricopeptide repeat-containing protein
FTR	*PDI* ^a^	4.43	VIT_201s0127g00560	probable nucleoredoxin 1-like
FTR	*HSP17II*	4.01	VIT_204s0008g01590	17.3 kDa class ii heat shock protein
FTR	***HSP20***	3.99	VIT_209s0002g06790	hsp20-like chaperone
FTR	*cHSP21*	5.56	VIT_216s0098g01060	chloroplast low molecular weight heat shock protein
TN_W	*E12A11*	1.82	VIT_200s0203g00080	protein mother of ft and tf 1
TN_W	*LEA*	1.81	VIT_203s0038g04390	late embryogenesis abundant
TN_W	*HAI1_HAI3*	1.78	VIT_206s0004g05460	protein phosphatase 2c
TN_W	*Lipase* ^a^	1.32	XM_002265963	GDSL esterase/lipase
TN_L	**PRP-I** ^a^	3.09	VIT_202s0154g00320	14 kDa proline-rich protein
TN_L	*NitTrans* ^a^	2.61	VIT_217s0000g09470	nitrate transmembrane transporter
TN_L	*PRP-II* ^a^	1.89	VIT_202s0154g00300	14 kDa proline-rich protein
TN_L	*GLP4*	1.86	VIT_217s0000g05360	rhicadhesin receptor
TN_L	*GA2OX8*	1.79	VIT_210s0116g00410	gibberellin 20-oxidase
TN_H	*CML44*	3.31	XM_002285850	probable calcium-binding protein CML44
TN_H	**ELIP1**	2.96	VIT_205s0020g04110	early light-inducible protein
TN_H	*GolS1*	2.64	VIT_207s0005g01980	galactinol synthase
TN_H	*DnaJ* ^a^	2.52	VIT_214s0060g01490	heat shock protein binding
TN_H	*ProKin* ^a^	2.19	VIT_218s0166g00010	probable LRR receptor-like serine/threonine-protein kinase
FTN	*PRP1* ^a^	3.58	VIT_211s0052g01650	pathogenesis-related protein
FTN	*BGLU17*	4.48	VIT_213s0064g01660	beta-glucosidase 13-like
FTN	*FTSH6*	3.76	VIT_214s0108g00590	cell division protease ftsh-6
FTN	*CoCHA* ^a^	3.66	VIT_203s0038g02110	chaperone protein dnaj chloroplastic-like
FTN	*Ankyrin* ^a^	3.62	VIT_205s0029g01410	ankyrin repeat-containing protein

^a^ Genes whose short name was attributed by the authors to facilitate writing. The five most up-regulated genes in Trincadeira (TR) and Touriga Nacional (TN) individual stress treatments (IS): Water deficit (W); High light (L); Heat (H); and Field: Trincadeira (FTR), Touriga Nacional (FTN). Name, expression value (in log_2_ gene expression, ID from 12x_v2.1 (http://genomes.cribi.unipd.it/grape/) or NCBI accession. Genes highlighted in bold represent genes that are shared between treatments and genes highlighted in underline represent genes shared between varieties, in Table 2 and between Table 2 and 3

**Table 3:** Down-regulated genes

Variety/Treatment	Name	Log_2_	ID	Annotation
TR_W	*LipGDSL* ^a^	−3.95	XM_002272934	GDSL esterase/lipase
TR_W	*VviEXPA18*	−3.92	VIT_217s0053g00990	expansin
TR_W	*CML* ^a^	−3.73	VIT_218s0001g01630	ef hand family protein
TR_W	*THI1*	−3.49	VIT_210s0116g00530	thiazole biosynthetic enzyme
TR_W	*PG2*	−3.47	VIT_201s0127g00850	probable polygalacturonase non-catalytic subunit jp650-like
TR_L	*Clmd* ^a^	−3.58	XM_002277463	putative calcium-binding protein CML19
TR_L	*ERF5-1*	−3.58	VIT_216s0013g00950	ethylene-responsive transcription factor 5
TR_L	**NUDT17**	−3.56	VIT_217s0000g02050	Nudix hydrolase 17
TR_L	*BAP2* ^a^	−3.19	VIT_215s0048g02070	BON1-associated protein (BAP2)
TR_L	*ASP*	−3.17	VIT_218s0001g07340	aspartyl protease
TR_H	*STZ*	−4.38	VIT_218s0001g09230	zinc finger protein ZAT10-like
TR_H	*WRKY46*	−4.15	VIT_215s0046g01140	WRKY transcription factor 46
TR_H	*SRS2*	−3.87	VIT_216s0013g00300	ATP-dependent DNA helicase
TR_H	*ERF-1*	−3.86	VIT_202s0234g00130	ethylene-responsive transcription factor 1a
TR_H	*CYP707A1*	−3.85	VIT_202s0087g00710	abscisic acid 8-hydroxylase
FTR	*SCPL7_SCPL18*	−4.04	VIT_203s0091g01290	serine carboxypeptidase-like 18-like
FTR	**PRP-I** ^a^	−3.64	VIT_202s0154g00320	14 kDa proline-rich protein
FTR	*GA20OX1*	−3.32	VIT_216s0022g02310	gibberellin 20-oxidase
FTR	*TT4*	−3.32	VIT_205s0136g00260	Chalcone synthase
FTR	*SCPL16_SCPL17*	−3.18	VIT_203s0088g00260	serine carboxypeptidase-like 18-like
TN_W	*THI1*	−3.49	VIT_210s0116g00530	thiazole biosynthetic enzyme
TN_W	*Pepd* ^a^	−1.54	VIT_218s0001g00510	prolyl oligopeptidase-like protein
TN_L	*HSP17*	−4.83	VIT_213s0019g02760	17 kDa class i heat shock protein
TN_L	*ERF5-1*	−3.62	VIT_216s0013g00950	ethylene-responsive transcription factor 5
TN_L	*HSP17II*	−3.43	VIT_204s0008g01500	17 kDa class ii heat shock protein
TN_L	*Clmd* ^a^	−3.58	XM_002277463	putative calcium-binding protein CML19
TN_L	*HSP18*	−3.41	VIT_213s0019g02770	18 kDa class i heat shock protein
TN_H	*SsP* ^a^	−4.36	VIT_200s0586g00030	stem-specific protein
TN_H	*XTR6*	−3.30	VIT_211s0052g01260	probable xyloglucan endotransglucosylase hydrolase protein
TN_H	*JAZ8*	−3.16	VIT_210s0003g03810	protein tify 5a-like
TN_H	**NUDT17**	−3.56	VIT_217s0000g02050	Nudix hydrolase 17
TN_H	*VviVQ3*	−2.87	VIT_202s0025g01280	WRKY transcription factor 41
FTN	**PRP-I** ^a^	−3.64	VIT_202s0154g00320	14 kDa proline-rich protein
FTN	*PYL4*	−3.41	VIT_213s0067g01940	abscisic acid receptor pyl4
FTN	*PRP-36* ^a^	−3.37	XM_003631687	36.4 kDa proline-rich protein
FTN	*PME61*	−3.17	VIT_205s0062g01160	pectinesterase family protein
FTN	*ZIP2*	−3.16	VIT_206s0004g05070	zinc transporter

^a^ Genes whose short name was attributed by the authors to facilitate writing. The five most down-regulated genes in Trincadeira (TR) and Touriga Nacional (TN) individual stress treatments (IS): Water deficit (W); High light radiation (L); Heat (H); and in the Field: Trincadeira (FTR), Touriga Nacional (FTN). Name, expression value (in log_2_ gene expression), ID from 12x_v2.1 (http://genomes.cribi.unipd.it/grape/) or NCBI accession. Genes highlighted in bold represent genes that are shared between treatments and genes highlighted in underline represent genes shared between varieties, in Table 3 and between Table 2 and 3

When Trincadeira (TR) plants were subjected to water stress (W) (Table 2) the gene *VviEXLB4*, an expansin precursor [26] with log_2_ (fold change) of seven was the most up-regulated gene (Table 2; Additional files 7 and 8). This gene family has been demonstrated to be highly expressed during the initial phase of drought stress in *Arabidopsis* [38, 39]. Conversely to TR, *VviEXLB4* was not significantly induced in TN, further evidence for the distinct response of this variety to drought. Other greatly up-regulated genes in TR under W include *VviMYBC2-L4* [40, 41] and *HSP20* both reported to be involved in several abiotic stresses including drought and heat [13, 29, 37]. In TR the five most up-regulated genes after light stress (L) were *OST1*, *ProOx*, *CXE*, *ZFC*, and *ELIP1* (Table 2; Additional files 7 and 8). *OST1* belongs to the serine threonine-protein kinase protein class and is involved in response to several abiotic stresses [42–44] and *CXE* codes for a protein with carboxylesterase activity with a role in plant detoxication [45]. In TR under heat stress (H) four of the five most up-regulated genes code for small heat shock proteins (HSPs) (Table 2 and Additional file 7) confirming this family as the most important class of genes responding to heat stress [8, 13]. The fifth gene, *GolS1*, belongs to the galactinol synthase family previously described as responsive to drought and dehydration [46, 47], as well as, induced by heat in grapevine berries [48, 49]. Regarding the most down-regulated genes (Table 3), in TR under W stress, two genes coding for cell wall remodelling enzyme were significantly repressed: the expansin precursor (*VviEXPA18*, conversely to the up-regulated *VviEXLB4* referred to above) and a polygalacturonase gene (*PG2*). Among the down-regulated transcripts after W stress were a lipase, a *CML* and a gene coding for a thiazole biosynthetic enzyme (*THI1*) (Additional file 7). This gene was described as playing an important role in mitochondrial DNA damage tolerance, [50–52], membrane modulation [53], and as being over-expressed under low temperature conditions [37]. The fact that our plants were kept at room temperature (22–25 °C) except in H treatment, can explain the down-regulation of this gene after the H of IS treatment. Under L stress, the most down-regulated genes were assigned to four functional categories: Unknown (*BAP2*), Metabolism (*ASP* and *NUDT17*), Signalling (*Clmd*) and Regulation (*ERF5-1*) [54, 55] (Additional file 7). *Clmd* codes for calmodulin, involved in signaling pathways through the modulation of the activity of other enzymes [56]. Under H the most down-regulated genes include the ABA hydrolase *CYP707A1*, one ethylene responsive factor (*ERF-1*), a zinc finger ZAT10 (*STZ),* one cytosolic class-I small heat-shock protein *(HSP18)* and *WRKY46* (Additional file 7). In field NI conditions (F) the most down-regulated genes in TR were within the Metabolism functional category, two serine carboxypeptidase (*SCPL7_scpl18* and *scpl16_scpl17*), one chalcone synthase (*TT4*), a gibberellin oxidase (*GA20OX1*) and a fifth transcript of the Transport functional category that codes a 14 kDa proline-rich protein also down-regulated in Touriga Nacional field NI (Additional file 7).

Focusing on Touriga Nacional (TN) plants, due to the low magnitude of change obtained in the microarray across the several ISs, some of the five most up (Table 2), or down regulated genes (Table 3) have log_2_ (fold change) < 2. Within the four most up-regulated genes is a late embryogenesis abundant protein transcript (LEA) which is involved in dehydration and desiccation [57, 58]. Under L, the five most up-regulated genes belong to the Transport or Metabolism (Primary and Secondary) functional categories (Additional file 7). Under H, the induced genes have all log_2_ (fold change) > 2, *CML44*, *ELIP1* (as in TR L) and *GolS1* (as in TR H), the HSP gene *DnaJ* and *ProKin*, a gene coding a protein kinase (Additional file 7). Of note is the absence of small HSPs.

Considering the TN down-regulated genes (Table 3) in water (W) stress, only two transcripts fulfilled the established criterion, one being *THI1*, also a down-regulated gene in TR after W. It is interesting to note that the most down-regulated genes found after light (L) are three HSPs, an ERF and a calcium-binding protein (Clmd), both in common with TR L. Under H the most down-regulated genes include *XTR6* a gene for a remodelling cell wall protein, the gene for the same Nudix hydrolase (*NUDT17*) referred to above as down-regulated in TR under L stress, and the gene *VviVQ3*, coding for a WRKY-interacting factor [23], that may be implicated in the response to biotic stress in *Arabidopsis* [59]. Although this *WRKY* gene isoform was down-regulated in TN under H, other *V. vinifera* cv. Aragonez WRKY transcription factors responded to drought and heat stress [8]. In TN, the most-responsive genes to the NI field treatment showed changes in expression ranging between 3.6 and 4.5 log_2_ (fold change). The most up-regulated genes were the biotic stress responsive proteins (*PRP1*) and ankyrin repeats protein [60] a β-glucosidase (*PHA*) a cell division protease (*FTSH6*) and a co-chaperone HSP (*CoCHA*). The five most down-regulated genes code for an ABA receptor (*PYL4*), a pectinesterase, a Zn transporter and two proline-rich proteins (*PRP-I* and *PRP-36*) (Additional file 7). Curiously, (*PRP-I)* was up-regulated after L stress as referred above.

### Array validation by RT-qPCR

The expression of the five most up- and down-regulated genes selected from the microarray (Tables 2 and 3) were quantified by RT-qPCR in individual stress (IS) and field (F) samples (Additional file 8) in order to assess the correlation between both methods. The obtained correlation coefficients were above 0.9 except for Touriga Nacional (TN) under water deficit (W) stress (*r* = 0.75) and high light (L) stress (*r* = 0.57) (Additional file 9). In general expression trends measured by microarray and RT-qPCR were the same with few exceptions. Although in Trincadeira (TR) *VviMYBC2-L4* was up-regulated by W in the array, it was down-regulated in the RT-qPCR. The four up-regulated transcripts in TN W were down-regulated in the RT-qPCR (Additional file 8). One possible explanation regarding the discrepancy of these up-regulated genes is the extremely low log_2_ (fold change) values, just over the limit value, of TN W genes, which barely fulfilled the established criteria.

### Except for drought, less transcriptome reprogramming is activated in Touriga Nacional under combined abiotic stresses

**Fig. 4:**
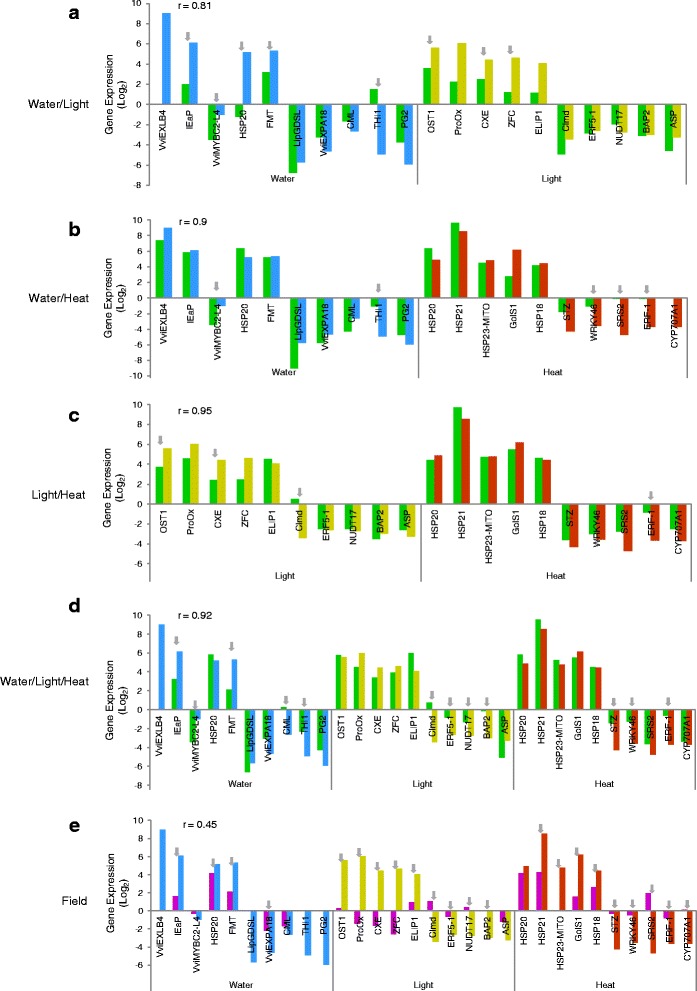
Gene expression response upon combined abiotic stresses in Trincadeira. Gene expression analysed by RT-qPCR for the five most up- and down-regulated genes when ISs are combined: water:high light (**a**); water:heat (**b**); high light:heat (**c**); water:high light:heat (**d**); field conditions (**e**). Colour-code for gene expression according to stress response: water deficit (blue); high light (yellow); heat stress (red); the combination of water deficit, high light and/or heat (dark green) and genes expressed in the field experiment (purple). Grey arrows represent significant statistical differences in gene expression between individual and combined stress treatments under controlled conditions or field experiment, *p* < 0.05. Log_2_ gene expression was normalized to control conditions. Gene annotation as in Tables 1 and 2. r, correlation coefficient

**Fig. 5:**
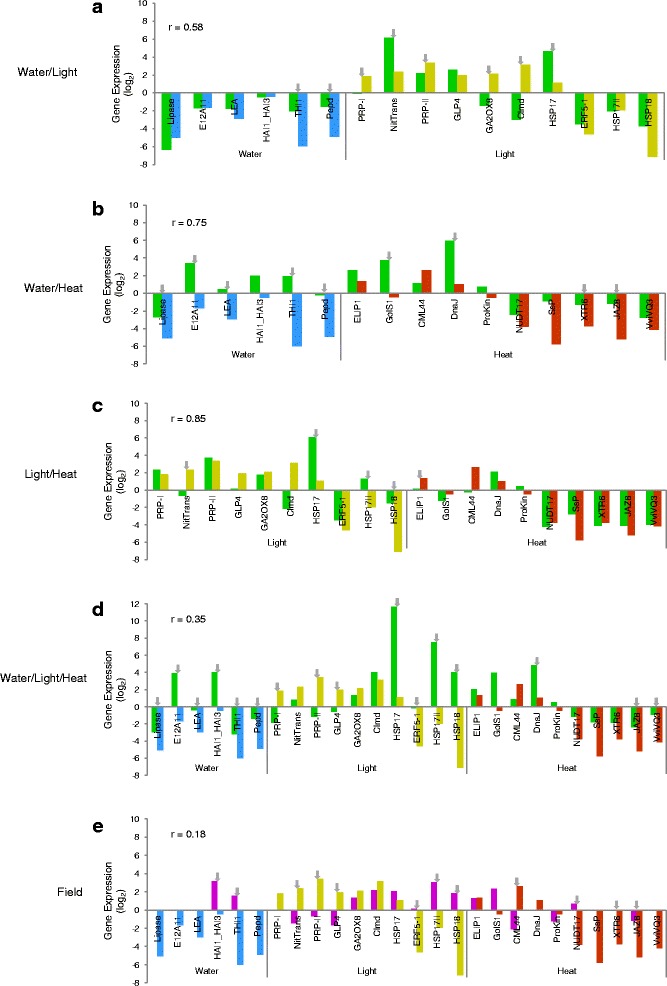
Gene expression response upon combined abiotic stresses in Touriga Nacional. Gene expression analysed by RT-qPCR for the five most up- and down-regulated genes when IS are combined: water:high light (**a**); water:heat (**b**); light:heat (**c**); water:high light:heat (**d**); field conditions (**e**). Colour-code for gene expression according to stress response: water deficit (blue); light stress (yellow); heat stress (red); the combination of water deficit, light and/or heat (dark green) and genes expressed in the field experiment (purple). Grey arrows represent significant statistical differences in gene expression between individual and combined treatments under controlled conditions or field experiment, *p* < 0.05. Log_2_ fold change was normalized to control conditions. Gene annotation as in Tables 1 and 2. r, correlation coefficient

The expression of the genes presented in Tables 2 and 3 was analysed in the leaf samples collected under combined stress treatments [water;high light (WL), water;heat (WH), high light;heat (LH) and water;high light;heat (WLH)] by RT-qPCR To ascertain the genomic response of field (F) NI samples, Trincadeira (TR) and Touriga Nacional (TN) leaves were probed as well (Fig. 4 and 5). The actual experimental approach is rather unique so few results are available so far, only allowing a scarce comparison with our data. Considering TR, the correlation between the values in the respective ISs (W and L) and the combined WL was 0.81. The combined water;high light treatment attenuated the response observed in W for several genes (*VviEXLB4*, *IEaP*, *HSP20*, *FMT*, *THI1*) indicating that water responsive genes, including those previously reported [9, 13], have their expression inversed when W and L stresses are imposed simultaneously (Fig. 4a). In contrast WL enhanced the response of several L up-regulated genes (*OST1*, *CXE*, *ZFC*) (Fig. 4a). Among the down-regulated genes in TR analysed by RT-qPCR, after WL stresses, only thiazole biosynthetic enzyme (*THI1*) changed its expression pattern. However, down-regulation of *THI1* seems to be countered by the simultaneous imposition of WL, becoming up-regulated. *THI1* response to W was also mitigated when W and H were combined (Fig. 4b), similarly to the response of H down-regulated genes (*WRKY*, *SRS2*, *ERF-1* and *CYP707A1*), although the overall correlation was very high, 0.9. Under LH (Fig. 4c) the response of three L-responsive genes (*OST1*, *CXE* and *Clmd*) and only one H-responsive gene (the H down-regulated *ERF-1*) were also significantly reverted. Similarly, a broad transcriptome inhibition after drought combined with heat had been shown before in *Arabidopsis thaliana* plants [9, 61], indicating a reversal of drought responses by heat. The lower down-regulation of *ERF-1* and *Clmd* in the combined LH might reveal more about their functions and interaction regarding environmental responses (see above). Of note is *V. pseudoreticulata ERF-1* which showed contrasting transcriptional response to different abiotic stress treatments [62]. The low variations in Trincadeira genes after LH treatment made their overall correlation the highest of the tested combined stresses (*r* = 0.95) (Fig. 4c). This suggests most of the responses to L and H in TR are independent or similar as reported for *Arabidopsis*, even though combined high light and heat response only correlated strongly with the heat response [63]. When the three individual stresses were combined (WLH) (Fig. 4d) the expression of most W-responsive genes was significantly attenuated. High light (L) and heat (H) up-regulated genes showed the same profile under WLH. Where a lower response of down-regulated genes under IS was generally observed, the overall correlation was still high, 0.92 (Fig. 4d). In TR field (Fig. 4e) samples, many W-responsive genes did not amplify, while the response of most other IS-selected genes decreased with exception of the W and L down-regulated *CML*, and *ASP*, respectively, and the consistently up-regulated *HSP20* upon H stress, a low correlation of 0.45 was obtained.

Considering Touriga Nacional (TN), when W and L stresses were combined (Fig. 5a) the expression of W- and L-responsive genes changed significantly. Down-regulation of *THI1* and *Pepd* by W was significantly attenuated and L-responsive transcripts exhibited a higher variation. The up-regulated response of *NitTrans* and *HSP17* was enhanced under WL, while the response of four other L up-regulated genes was attenuated or reversed resulting in *r* = 0.58 (Fig. 5a). WH combination gave rise to an even higher variation in the W responsive transcripts (Fig. 5b). Considering H-responsive transcripts, the expression of two up-regulated genes (*GolS1* and *DnaJ*) was enhanced while the down-regulation of *XTR6* and *JAZ8* was attenuated. However, the r value in WH was 0.75 (Fig. 5b). Gene expression after LH (Fig. 5c) was consistent with the respective individual stress RT-qPCR values with an *r* = 0.85, the highest in TN stress combinations (Fig. 5c). Touriga Nacional results confirm that responses to L and H follow the same trend as in TR (above) and *Arabidopsis* [63]. After WLH treatment (Fig. 5d), all the W and the majority of the L response genes exhibit significantly different expression profiles, while H-responsive genes maintained the same trend. The variation in W and L transcripts caused a low r, 0.35 (Fig. 5d). In TN field NI samples, when the IS transcripts were tested most transcripts did not amplify or their expression was significantly different from IS gene expression, resulting in a very low r value, 0.18 (Fig. 5e).

Our results confirm the limited accuracy of extrapolating the effects of individual, or even combined, abiotic stress in controlled conditions to actual plant growth and functioning [10]. Grafted and well rooted grapevine plants growing in the field or potted plants obtained from cuttings in controlled conditions certainly respond differently to environmental changes. Taken together, the above results show how complex the relationship is between gene expression and the environmental setting. However, the experimental set up applied to two genotypes can elucidate unique transcriptomic responses associated with particular tolerance mechanisms after each stress, per variety. Furthermore, our results also show that the grapevine capacity to manage abiotic stress response is intrinsically variety-linked. While TR responded clearly to IS treatments and that response was maintained under combined ISs, in TN, the responses varied a great deal under IS combination, especially when W was involved. This seems to confirm the higher tolerance of TN, with less transcriptome reprogramming except when water deficit is involved [8, 13].

### Transposable elements and grapevine abiotic stress responses

It is known that most genomes comprise transposable elements (TEs) whose activation by stress suggests their role as key players in genome plasticity to survive in adverse environments [64, 65]. Grapevine is not an exception with about one fifth of its genome comprising this type of repeats [66]. To gain more information about the *Vitis* transposable sequences still not characterized, we queried those sequences present in the Unknown functional category against a transposable elements database (http://www.girinst.org/). In this query we obtained homologies for 20 % of the sequences with known transposable elements (Additional file 10: Figure S1) and we found that TEs are represented within Trincadeira (TR) and Touriga Nacional (TN) stress-responsive genes. The percentage of matches ranged from 28 to 40 % in TR and from 25 % to 36 % in TN (Additional file 10: Table S1). Within TEs, two classes are predominant, DNA transposons and LTR retrotransposons (Additional file 10: Tables S2 and S3).

Clustering the profiles of the expressed TEs using the k-means algorithm resulted in 8 groups in TR (Additional file 10: Figure S2) and 7 groups in TN (Additional file 10: Figure S3). These profiles showed that in Trincadeira W stress, 58.8 % of the TEs were down-regulated and 41.2 % were up-regulated while after L more or less the same number of up and down-regulated TEs was obtained. Strikingly, monitoring TEs in field Trincadeira samples (FTR) revealed a burst of activity with 70 % of the TEs up-regulated. The comparison between TR and TN under IS was somewhat compromised given the generally low activity in the latter. However, as in TR, 58.7 % TEs in field Touriga Nacional (FTN) showed up-regulation. Although detecting TEs transcripts does not necessary mean that they are transposing, we do not rule out the stress-induced TE mobility hypothesis put forward for other species [67, 68] favouring the genetic variability eventually useful for adaption to a changeable environment [65, 69]. Our data revealed several fragments of TEs (Additional file 10:Figures S4 and S5) that could partially contribute to the plasticity of individual genes, for instance, through new introns, exons, or chimeric genes. These are not rare events, since a high proportion of Angiosperm genes harbour TEs, as referred for rice where more than 10 % of transcripts are reported to contain TEs [70]. We also performed an expression profile analysis of the TEs common to the applied treatments (Additional file 10: Figure S6). The results revealed changes in the expression of some TEs, pointing to stress-induced TE activity, a result that can explain the adaptation to environmental challenges [69, 71]. The mechanisms involved in activation and repression of TEs in plants are still not clearly understood and are beginning to be unravelled. Our data show that TEs changed expression in a stress-specific manner, suggesting a potential role of TEs in grapevine stress response and adaptation, deserving further investigation.

## Conclusions

Microarray data were obtained from leaves of two grapevine varieties commonly used in Portugal for wine making, Touriga Nacional (TN) and Trincadeira (TR). They were subjected to individual abiotic stress treatments in growth room controlled conditions and in agronomic field conditions, and confirmed TN as less responsive to abiotic stress imposed in the growth room than TR. Regarding the combination of individual abiotic stresses, the expression of selected genes was as expected from the individual stresses (ISs), with only a few genes exhibiting statistically different levels of expression, pointing to antagonist or in lower frequency synergistic interplay between the ISs. Only under the field environmental constraint, when both varieties were compared for full irrigation (FI) and no irrigation (NI) did Touriga Nacional exhibit an unequivocal transcriptomic response. This result may indicate that TN has a different threshold for the level of stress it can withstand before triggering a response. A high number of differentially expressed genes were assigned to the Unknown category [21]. It cannot be excluded that several key genes for grapevine stress response are still with non-described function. An interesting fact was that 20 % of the manually re-annotated Unknown category genes were assigned to mobile elements, a significant class in any genome, including grapevine [66]. Finally, the results allow the conclusions: 1) experiments in controlled conditions still remain a useful resource to study the effect of single stresses although correlations to field condition results must be cautious; 2) varieties of the economically important crop grapevine, even when cultivated in common areas under extreme environmental conditions, can display gene expression profiles evidencing a considerable intra-species diversity of responses to the environment; 3) mobile elements are well represented in grapevine stress-responsive transcriptome but their response to abiotic stress remains mostly unknown, deserving to be studied in depth.

## Methods

### Growth room plant material and stress treatments

Cuttings from pruned wood of selected *Vitis vinifera* L. plants of the varieties Touriga Nacional (TN) and Trincadeira (TR) were grown in 3 l pots in the growth room under the following controlled conditions: 200 μmol quanta m^−2^ · s^−1^ irradiance, 16 h light/8 h dark photoperiod, temperature of 25 °C day/ 23 °C night and well-watered with nutrient solution [72]. The growth room has 72 m2 and is adapted to provide controlled conditions of light and temperature.

Individual stresses (ISs) were applied when shoots were 50 to 60 cm high (after *circa* four months from cuttings planting) always in the middle of the light period, so that sampling took place shortly after that period. The treatments applied were: W – stop irrigation until the pre-dawn leaf water potential (Ψw) was −0.9 MPa, value that is consistent with a severe water stress [73]; high light (L) – 1 h at 2,000 μmol · m2 · s-1; heat (H) – 1 h at 42 °C (temperature measured at the surface of the leaf) provided by a homogenous heat source; WL – a combination of both L and W; WH – a combination of both W and H; LH – a combination of L and H; WLH – a combination of W, L and H. Ψw was measured with a pressure chamber, Model 600, PMS Instruments Company (Albany, OR). Samples consisted of the first, second and third totally expanded leaves from the shoot apex and were taken 1 h after the start of the stress treatment together with control samples (or, in the case of WS, when the pre-dawn leaf Ψ_w_ reached the desired value), frozen in liquid nitrogen and stored at −80 °C until RNA extraction. Chlorophyll a fluorescence was measured using a Pulse Amplitude Modulation Fluorometer (mini-PAM, Photosynthesis Yield Analyzer Walz, Germany), with a saturation pulse intensity extending up to 18000 μmol photons m-2 s-1 and actinic light corresponding to the Photosynthetically Active Radiation (PAR). Dark adaptation for chlorophyll a fluorescence was performed and the following measurements were undertaken: ground state fluorescence (Fo), maximal fluorescence (Fm), variable fluorescence (Fv = Fm – Fo) and maximum quantum yield of the PSII system (Fv/Fm = [Fm – Fo]/Fm); together with the light adapted measurement of F’v/F’m.

### Field plant material and irrigation treatment

The field trial plants, Touriga Nacional and Trincadeira, were from the grapevine selection collection, PORVID Association, Pegões, PT (38° 38’ 55 N; −8° 39’ 14 O).

The climate in the Pegões vineyard is Mediterranean with Atlantic influence with hot dry summer days and fresh nights, and mild winters (Additional file 1). The soil is derived from podzols, with a sandy surface layer (0.6–1.0 m) and clay at 1 m depth. Several genotypes of Touriga Nacional and Trincadeira were established in the vineyard. Both varieties were grafted on 1103 Paulsen rootstock in 2002. The plants are spaced 2.5 m between rows and 1 m within rows and trained on a vertical trellis with a pair of movable foliage wires for upward shoot positioning. In the field, only irrigation was used as treatments, thus two treatments were applied: full irrigated (FI) and non- irrigated (NI). Irrigation water in FI treatment was applied with drip emitters (4.0 L h^−1^ for FI) two per vine, positioned 30 cm from the vine trunk. The water was supplied according to the crop’s evapotranspiration (ETc.). Samples were taken simultaneously in both varieties when the pre-dawn leaf water potential was *circa −*0.7 MPa in the NI plants and higher than −0.2 MPa in the FI plants (Fig. 1). Samples consisted of the first and second totally expanded leaves, per plant and per treatment, which were frozen in liquid nitrogen until RNA extraction.

### RNA extraction

Samples were ground with a mortar and pestle in the presence of liquid nitrogen. Total RNA was extracted using the Spectrum™ Plant Total RNA kit (Sigma-Aldrich, St. Louis, MO, USA). RNA quality and quantity were determined using a Nanodrop 2000 spectrophotometer (Thermo Scientific, Wilmington, DE, USA).

### Hybridization to Affymetrix GeneChips

Thirty-six samples (three replicates per treatment including control plants) were analysed at the Genomics Unit of the Spanish National Centre for Biotechnology (CNB-CSIC, Madrid). RNA integrity analyses were done with an Agilent’s Bioanalyzer 2100 using the NanoChip protocol [8]. The custom GrapeGen Affymetrix GeneChip® (A-AFFY-162 and GPL11004 ArrayExpress and GEO accession numbers, respectively) [24], was processed as previously described [74]. Briefly, biotinylated RNA was prepared from 2 μg of total RNA according to the standard Affymetrix protocol. After the first and second strand synthesis of cDNA in vitro transcription was performed using T7 RNA polymerase and biotinylated nucleotides, to produce biotin-labeled cRNA. Labelled cRNA was fragmented to the 50–200-bp size range, and each sample was added to a hybridization solution containing 100 mM 2-(N-morpholino) ethanesulfonic acid, 1 M Na+, and 20 mM of EDTA in the presence of 0.01 % of Tween-20 to a final cRNA concentration of 0.05 μg/ml. Hybridization was performed for 16 h at 45 °C [8]. Each GeneChip was washed and stained with streptavidin-phycoerythrin in a Fluidics Station 450 (Affymetrix) following the EukGE-WS2v5 script. After washing, the chips were scanned at 1.56 μm resolution in a GeneChip® Scanner 3000 7G System (Affymetrix). The software used was GeneChip Operating Software.

### Data analysis

Eight data arrays from a total of 36 samples analysed (eight sample types in three biological replicates) were normalized to a baseline array with median CEL intensity by applying an Invariant Set Normalization Method [75]. Normalized CEL intensities of the arrays were used to obtain model-based gene expression indices based on a PM (Perfect Match)-only model [76]. Replicate data (triplicates) were weighted gene-wise by using inverse squared standard error as weights and analysed using the DNA-Chip Analyzer software. It allows assessment for expression indexes and calculation confidence intervals for fold changes. A lower confidence bound (LCB) cutoff between 1.2 and 1.3 was used to assess differentially expressed genes with a median False Discovery Rate (FDR) < 5 % [76].

All transcripts were annotated following 12x_v2.1 draft annotation of the grapevine genome [21] allowing 70 % of the genes to be identified within the 12x_v2.1 assembly reference genome. A gene was declared to be differentially expressed in a given condition only when it had a presence call in all three replicates. The subsequent validation of this approach was performed by RT-qPCR.

Microarray data analysed in this study have been submitted to the Gene Expression Omnibus (GEO) database (www.ncbi.nlm.nih.gov/geo/) under the number GSE57669.

### Principal component analysis (PCA)

PCA projects high-dimensional data into lower dimensions by summarizing the variation of a high number of variables to a reduced number of principal components, and calculates the proportion of variation explained by each principal component [77]. Individual replicates of samples were projected into a three-dimensional space (http://www.partek.com/pgs) according to their expression data. The first three principal components were plotted.

### cDNA synthesis for RT-qPCR

Nucleic acid concentration of each sample was quantified by spectrophotometry using the software Gen5 1.09 (Synergy HT, Bio-Tek Instruments, Winooski, USA). Total RNA quality was assessed using the A_260_/A_280_ and A_260_/A_230_. Only RNA samples with A_260_/A_280_ between 1.8 and 2.1 and A_260_/A_230_ between 2.0 and 2.2 were used. Total RNA integrity was checked through 1 % agarose gel electrophoresis under denaturing conditions (data not shown). RNA samples were treated with RQ1 RNase-Free DNase (Promega, Madison, WI, USA). cDNA was synthesized from 2 μg of total RNA using oligo(dT)_20_ in a 20 μL-reaction volume using RevertAid Reverse Transcriptase (Fermentas Life Science, Helsingborg, Sweden) according to the manufacturer’s recommendations. cDNA was stored at −20 °C until further use.

### Gene selection for microarray validation and primer design

Genes for the microarray validation were selected from the five most highly up-regulated genes and five most highly down-regulated genes for each variety/treatment (log_2_ [treatment/control] fold change higher than 2 or lower than −2). Deviations from this principle occurred when there was no NCBI accession number for the selected gene plus its functional category and annotation was unknown/no hit or repeated in the other five selected genes. In these cases, subsequent genes were selected providing their expression (in log_2_) was higher/lower than 2/-2. In Touriga Nacional (TN) under water (W) and light (L) stress the amount of transcripts was small and there were few or none with expression above or lower than 2/-2 therefore the most up or down-regulated genes were chosen. This resulted in a total selection of 65 genes across varieties/treatments (32 up-regulated and 33 down-regulated). Primers for these genes were designed using the software Primer Premier 5.0 (Premier Biosoft International) using a primer length of 20 ± 2 bp, melting temperature of 60 °C ± 2 °C, a guanine-cytosine content of *circa* 50 % and an expected amplicon size of 180–280 bp (Additional file 11). We also checked, *in silico*, primer specificity compared to the grapevine 12x_v2.1 assembly reference genome (http://genomes.cribi.unipd.it/grape/).

### Real-Time qPCR

The primer selection for RT-qPCR is of the utmost importance [78]. So we adopted primers and microarray probes to target the same exon to avoid possible bias introduced by the secondary structure of the cDNA [79] or by differential splicing. Special attention was also paid to the selection of the internal references and normalization methods, since they can influence the results significantly [80]. The real-time qPCR was performed in 96 well white reaction plates (Bio-Rad, Hercules, CA), using an IQ5 Real Time PCR (Bio-Rad, Hercules, CA) with three biological replicates and two technical replicates. The 20 μL reaction mixture was composed of 1 μL cDNA diluted 50-fold, 0.5 μM of each gene-specific primer and 10 μL master mix (SsoFast_EvaGreen Supermix, Bio-Rad, Hercules, CA). Amplification of PCR products was monitored via intercalation of Eva-Green (included in the master mix). The following program was applied: initial polymerase activation, 95 °C, 3 min; then 40 cycles at 94 °C 10 s (denaturation), 60 °C 20 s (annealing), 72 °C 15 s (extension), followed by a melting curve analysis to confirm the correct amplification of target gene fragments and the lack of primer dimmers. The PCR products were run on 2 % agarose gels to make sure that there was only one amplicon of the expected size. PCRs with each primer pair were also performed on samples lacking cDNA template, in triplicate (no template controls). To assess amplification efficiency of the candidate genes, identical volumes of cDNA samples were diluted and used to generate five-point standard curves based on a five-fold dilution series (1; 1:5; 1:25; 1:125; 1:625), in triplicate. Amplification efficiency (E) is calculated as E = 10^(−1/a)^-1, “a” being the slope of the linear regression curve (y = a log(x) + b) fitted over the log-transformed data of the input cDNA dilution (y) plotted against the respective quantification cycle (Cq) values (x). E-values of the target genes were considered comparable when they did not exceed 100 ± 10 %, corresponding to a standard curve slope of 3.3 ± 0.33. All cDNA samples were diluted 50 fold and were amplified in duplicate in two independent PCR runs.

To generate a baseline-subtracted plot of the logarithmic increase in fluorescence signal (ΔRn) versus cycle number, baseline data were collected between cycles 5 and 17. All amplification plots were analysed with an *R*_*n*_ threshold of 0.2 at the beginning of the region of exponential amplification, to obtain Cq (quantification cycle) and the data obtained were exported into a MS Excel workbook (Microsoft Inc., USA) for analysis. Reference genes used were *ACT*, *TIF* and *TIF-GTP* [80].

### Statistical analysis of Real-time qPCR

For the relation between the expressions of the selected genes and the reference genes the relative quantity values were transformed into log_2_ (thus rendering them parametric) and tested through ANOVA in the program SAS 9 (for Windows, SAS Institute Inc., Cary, NC, USA). When the *p* value of the ANOVA was lower than 0.05 a Tukey test was performed and statistically significant differences were accepted for a *p* value lower than 0.05.

## Additional files

Additional file 1: Maximal, medium and minimal temperatures and precipitation at Pegões Experimental Station (38° 38’ 55 N; −8° 39’ 14 W). (PDF 223 kb)
Additional file 2: Hierarchical cluster analysis (HCA) of microarray results for both varieties, Trincadeira and Touriga Nacional. (PDF 63 kb)
Additional file 3: Gene expression responses shared between the two varieties under high light and heat. TN, Touriga Nacional; TR, Trincadeira; H, heat stress; L, high light stress. (XLSX 14 kb)
Additional file 4: Genes exclusive and shared between field and growth room controlled stress treatments. (PDF 157 kb)
Additional file 5: Distribution in functional categories of differentially expressed genes in growth room and field experiments. (PDF 302 kb)
Additional file 6: Percentage of genes expressed after Heat, Light and Water stress growth room treatments in Trincadeira and Touriga Nacional plants in three selected functional categories. (PDF 290 kb)
Additional file 7: Functional categories of the selected up-and down-regulated genes. (XLSX 19 kb)
Additional file 8: Validation of the microarray gene expression by RT-qPCR. (PDF 31 kb)
Additional file 9: Correlation between the gene expression from the microarray and RT-qPCR analysis. (PDF 141 kb)
Additional file 10: Analyses of responsive transcripts in the Unknown Functional Category. (PDF 453 kb)
Additional file 11: Primers used for amplification of up- and down- regulated genes by RT-qPCR. (XLSX 13 kb)

## Abbreviations

ABA: Abscisic acid
ACT: Actin gene
C: Control sample
°C: Degrees Celsius
ELIP: Early Light-Inducible Protein
FDR: False Discovery Rate
FI: Full irrigation
FTN: Field Touriga Nacional
FTR: Field Trincadeira
h: Hours
H: Heat
HCA: Hierarchical clustering analysis
IS: Individual stress
L: High light
LH: High light;heat
LTR: Long terminal repeats
m: Meter
MPa: Mega Pascal
NI: No irrigation
PCA: Principal component analysis
PC1: Principal component 1
PC2: Principal component 2
PSII: Photosystem II
RT-qPCR: Quantitative reverse transcriptase-polymerase chain reaction
TEs: Transposable elements
TF: Transcription factor
TIF: Translation initiation factor eIF-3 subunit 4
TIF-GTP: Translation initiation factor eIF-2B alpha subunit
TN: Touriga Nacional
TN H: Touriga Nacional Heat stress
TN L: Touriga Nacional High Light stress
TN W: Touriga Nacional Water stress
TR: Trincadeira
TR H: Trincadeira Heat stress
TR L: Trincadeira High Light stress
TR W: Trincadeira Water stress
W: Drought stress
WH: Water;heat stress
WL: Water;high light stress
WLH: Water;high light;heat stress
