# Supplementary material for: Transcriptomic comparison between two Vitis vinifera L. varieties (Trincadeira and Touriga Nacional) in abiotic stress conditions
Source: BMC Plant Biol. 2016 Oct 12;16:224. doi: 10.1186/s12870-016-0911-4 (PMC5062933; doi:10.1186/s12870-016-0911-4)
Supplement: Additional file 1: — Maximal, medium and minimal temperatures and precipitation at Pegões Experimental Station (38° 38’ 55 N; −8° 39’ 14 W). (PDF 223 kb) [file 12870_2016_911_MOESM1_ESM.pdf]

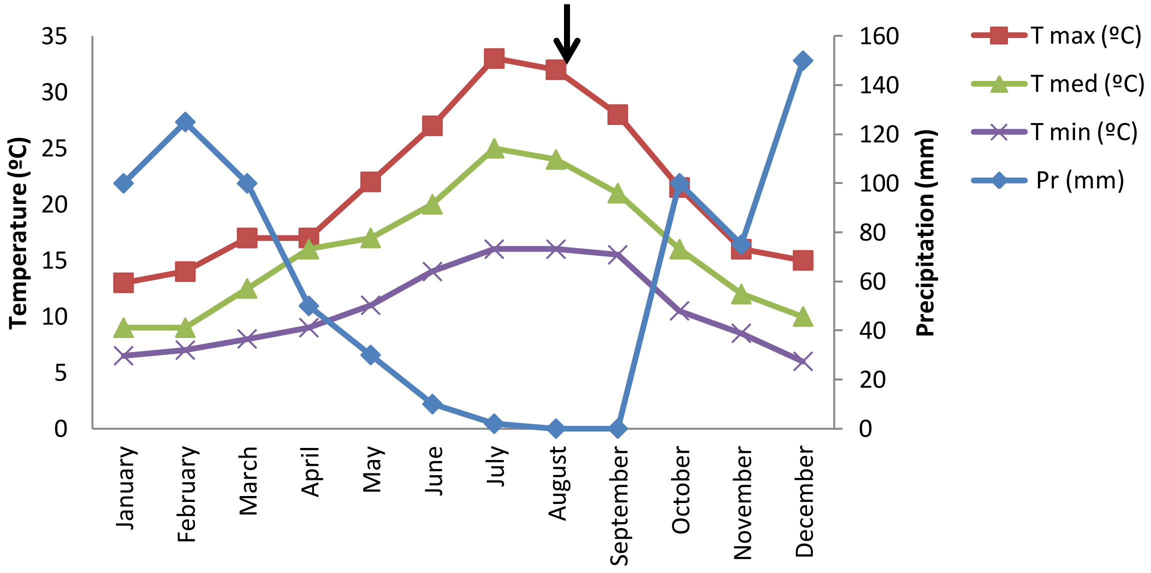

Additional file 1. Maximal, medium and minimal temperatures and precipitation at Pegões Experimental Station (38º 38’ 55 N; -8º 39’ 14 W). Temperature (in ºC) and precipitation (in mm) during 2010 season when the main experiment took place. Max temperature (red), average temperature (green) minimum temperature (purple) and precipitation (blue). Date of sampling (06th August) is indicated by the vertical black arrow.
