# Supplementary material for: Transcriptomic comparison between two Vitis vinifera L. varieties (Trincadeira and Touriga Nacional) in abiotic stress conditions
Source: BMC Plant Biol. 2016 Oct 12;16:224. doi: 10.1186/s12870-016-0911-4 (PMC5062933; doi:10.1186/s12870-016-0911-4)
Supplement: Additional file 2: — Hierarchical cluster analysis (HCA) of microarray results for both varieties, Trincadeira and Touriga Nacional. (PDF 63 kb) [file 12870_2016_911_MOESM2_ESM.pdf]

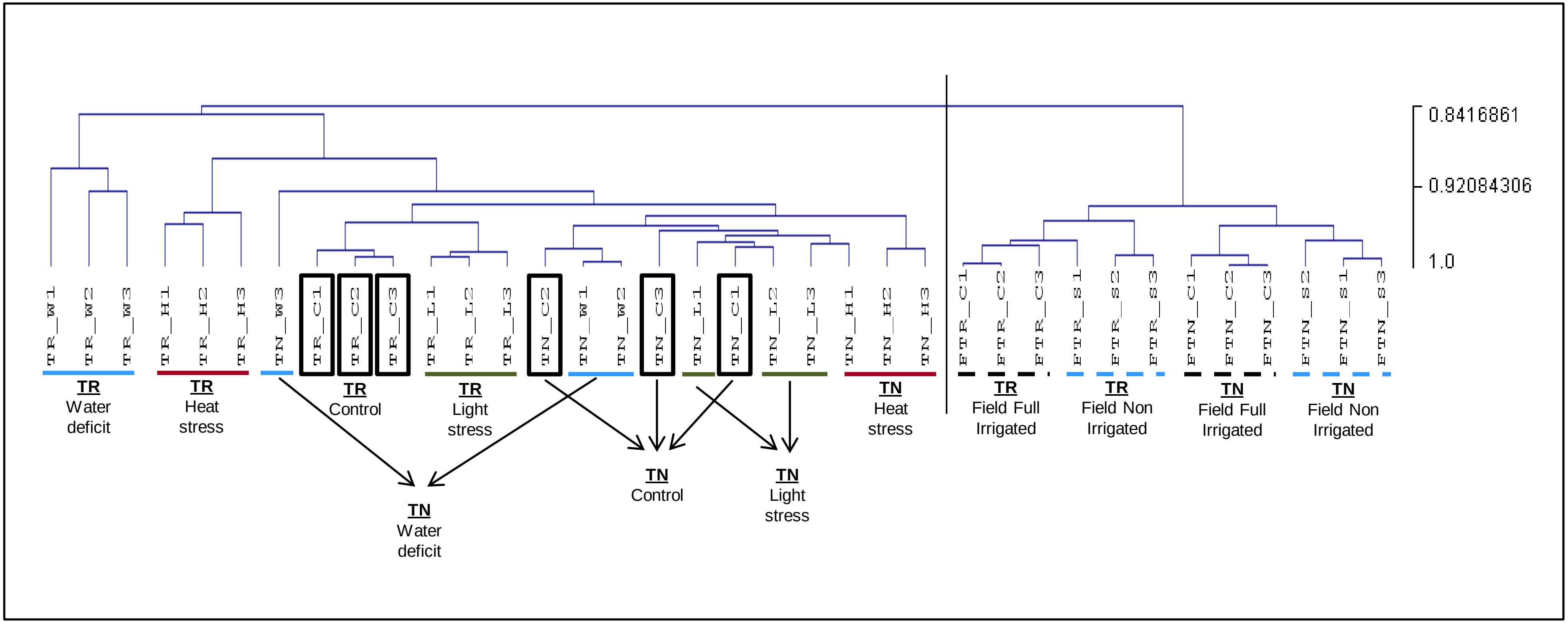

Additional file 2. Hierarchical cluster analysis (HCA) of microarray results for both varieties. Featuring all growth room individual stresses and field results. Trincadeira, (TR) and Touriga Nacional (TN); Growth room control (TN/TR, C - black boxes and lines); Water deficit (TN/TR, W – blue lines); High light radiation (TN/TR, L – green lines); Heat (TN/TR, H - red); Field full-irrigated (FTN/FTR, C - black dashed line); Field non-irrigated (FTN/FTR, NI - blue dashed line).
