# Supplementary material for: Transcriptomic comparison between two Vitis vinifera L. varieties (Trincadeira and Touriga Nacional) in abiotic stress conditions
Source: BMC Plant Biol. 2016 Oct 12;16:224. doi: 10.1186/s12870-016-0911-4 (PMC5062933; doi:10.1186/s12870-016-0911-4)
Supplement: Additional file 4: — Genes exclusive and shared between field and growth room controlled stress treatments. (PDF 157 kb) [file 12870_2016_911_MOESM4_ESM.pdf]

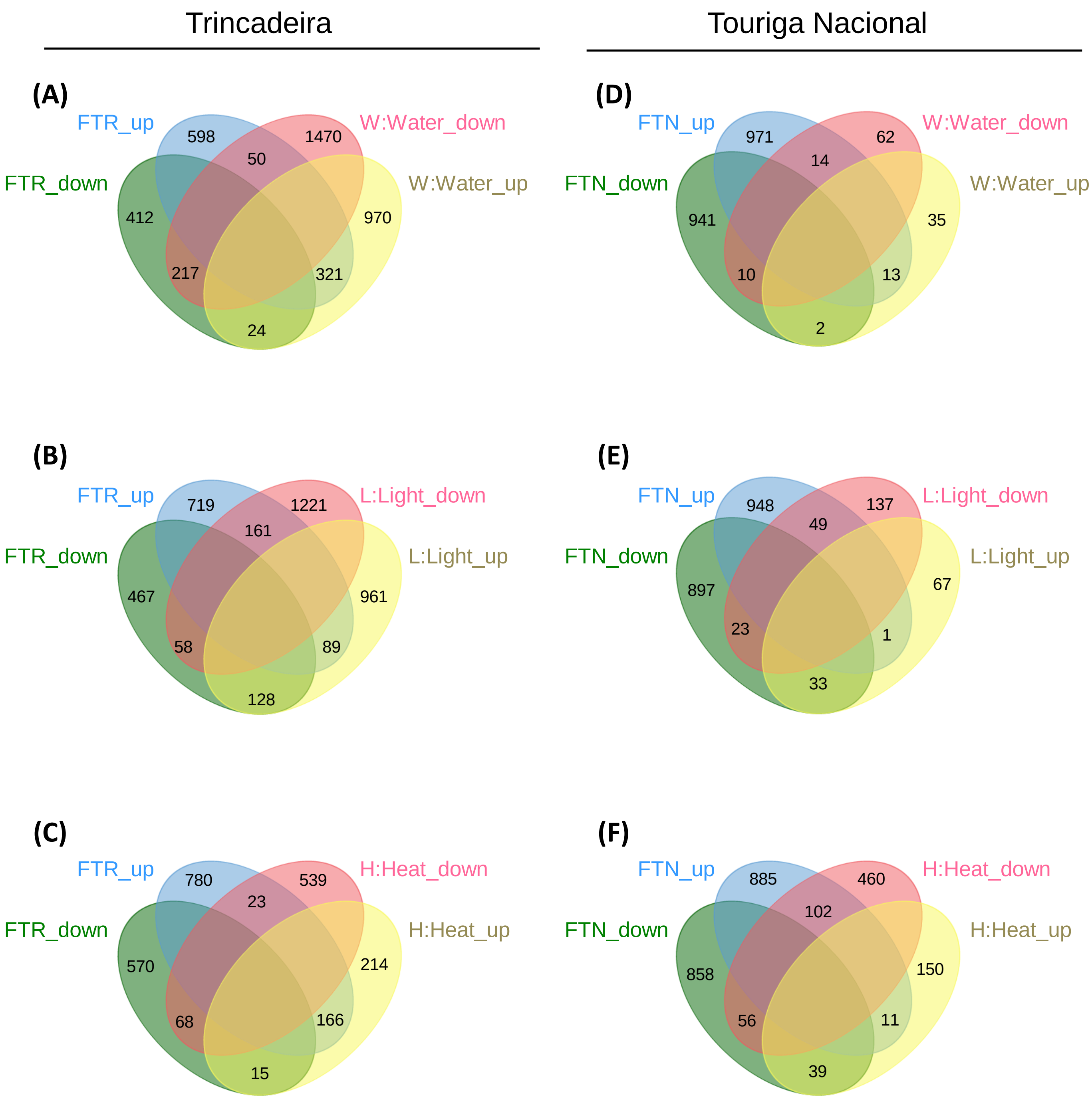

Additional file 4. Genes exclusive and shared between field and growth room controlled stress treatments. Diagrams show the distribution of the genes between Trincadeira (A, B and C) and Touriga Nacional (E, F and G). FTR, Field Trincadeira, FTN, Field Touriga Nacional. Up, up-regulated genes; Down, down-regulated genes.
