# Supplementary material for: Transcriptomic comparison between two Vitis vinifera L. varieties (Trincadeira and Touriga Nacional) in abiotic stress conditions
Source: BMC Plant Biol. 2016 Oct 12;16:224. doi: 10.1186/s12870-016-0911-4 (PMC5062933; doi:10.1186/s12870-016-0911-4)
Supplement: Additional file 5: — Distribution in functional categories of differentially expressed genes in growth room and field experiments. (PDF 302 kb) [file 12870_2016_911_MOESM5_ESM.pdf]

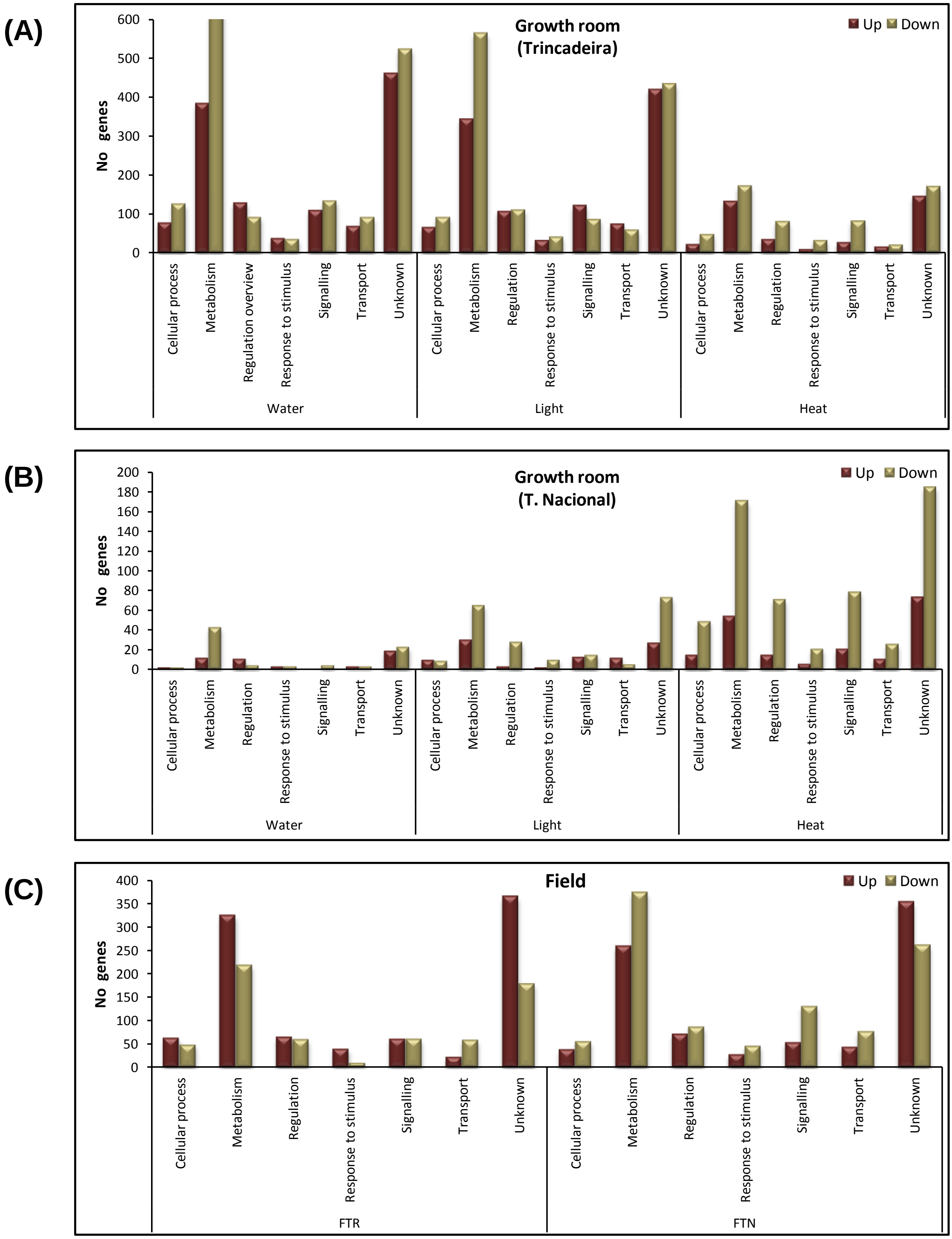

Additional file 5. Distribution in Functional Categories of differentially expressed genes in growth room and field experiments. Graphics show the distributions of expressed genes through functional categories. (A) Trincadeira response of individual growth room stresses. (B) Touriga Nacional (T. Nacional) response of individual growth room stresses. (C) Field response. Up, up-regulated genes; Down, down-regulated genes; FTR, Field Trincadeira, FTN, Field Touriga Nacional (T. Nacional).
