# Supplementary material for: Transcriptomic comparison between two Vitis vinifera L. varieties (Trincadeira and Touriga Nacional) in abiotic stress conditions
Source: BMC Plant Biol. 2016 Oct 12;16:224. doi: 10.1186/s12870-016-0911-4 (PMC5062933; doi:10.1186/s12870-016-0911-4)
Supplement: Additional file 6: — Percentage of genes expressed after Heat, Light and Water stress growth room treatments in Trincadeira and Touriga Nacional plants in three selected functional categories. (PDF 290 kb) [file 12870_2016_911_MOESM6_ESM.pdf]

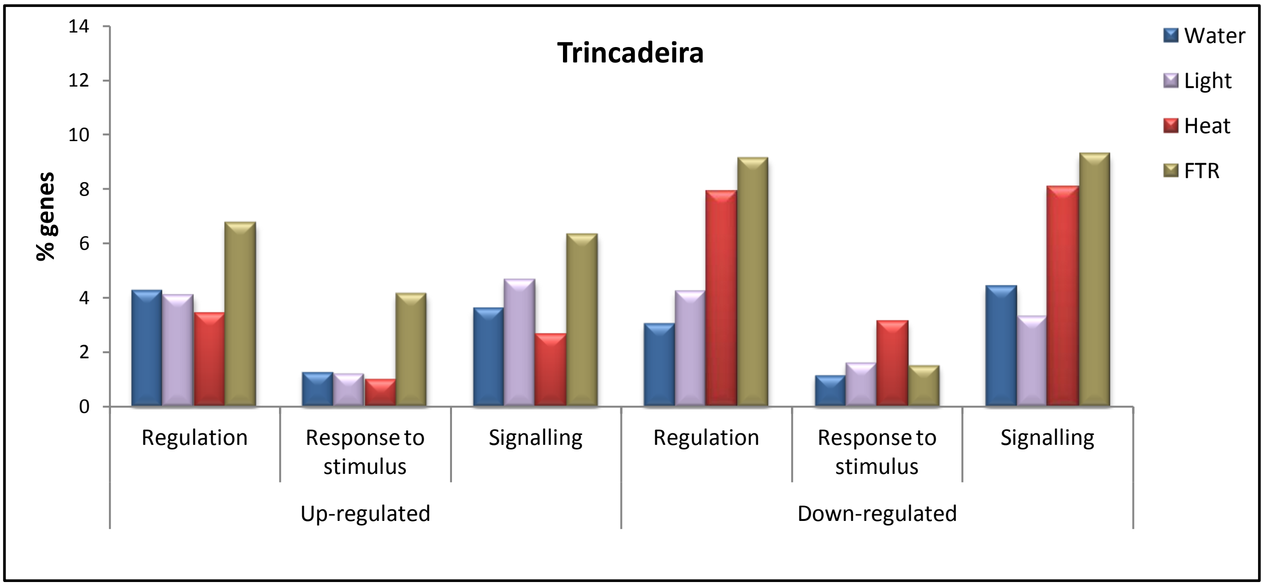

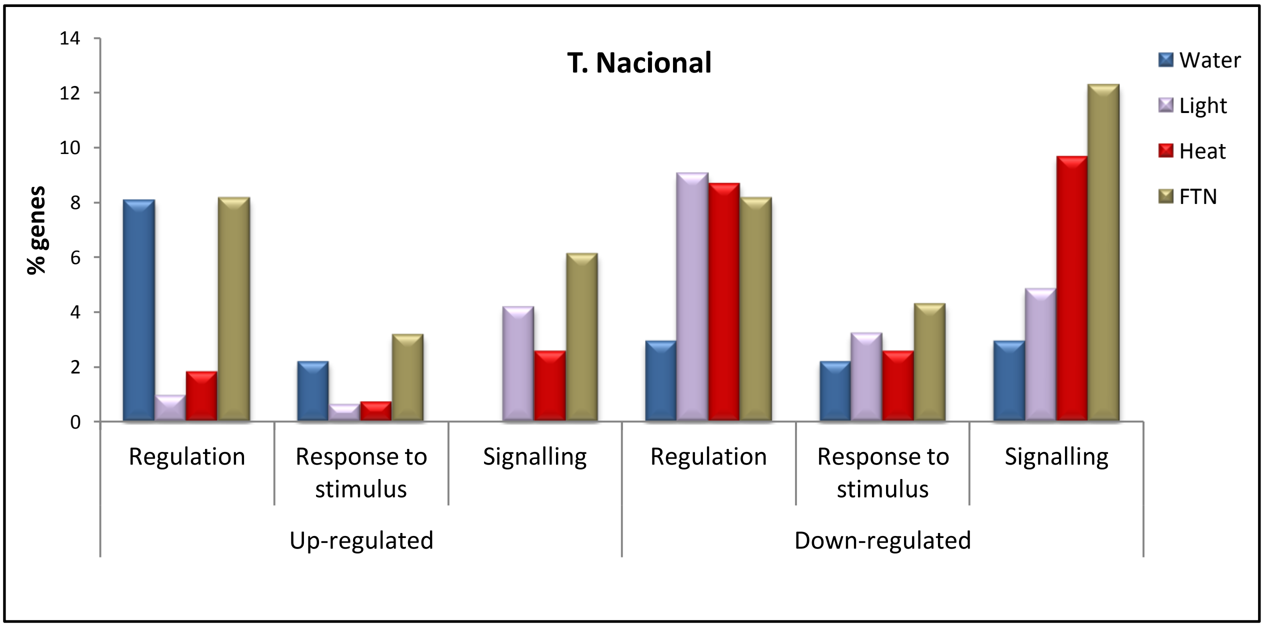

Additional file 6. Percentage of genes expressed after Heat, Light and Water stress growth room treatments in Trincadeira and Touriga Nacional plants in three selected functional categories. Detail overview of the up and down-regulated genes in the three most relevant categories by percentage of the total number of genes significantly responding to each stress. Response to stimulus category only includes Stress response genes and Regulation of transcription is a part of Regulation overview category. FTR, Field Trincadeira; FTN, Field Touriga Nacional (T. Nacional).
