# Supplementary material for: Transcriptomic comparison between two Vitis vinifera L. varieties (Trincadeira and Touriga Nacional) in abiotic stress conditions
Source: BMC Plant Biol. 2016 Oct 12;16:224. doi: 10.1186/s12870-016-0911-4 (PMC5062933; doi:10.1186/s12870-016-0911-4)
Supplement: Additional file 8: — Validation of the microarray gene expression by RT-qPCR. (PDF 31 kb) [file 12870_2016_911_MOESM8_ESM.pdf]

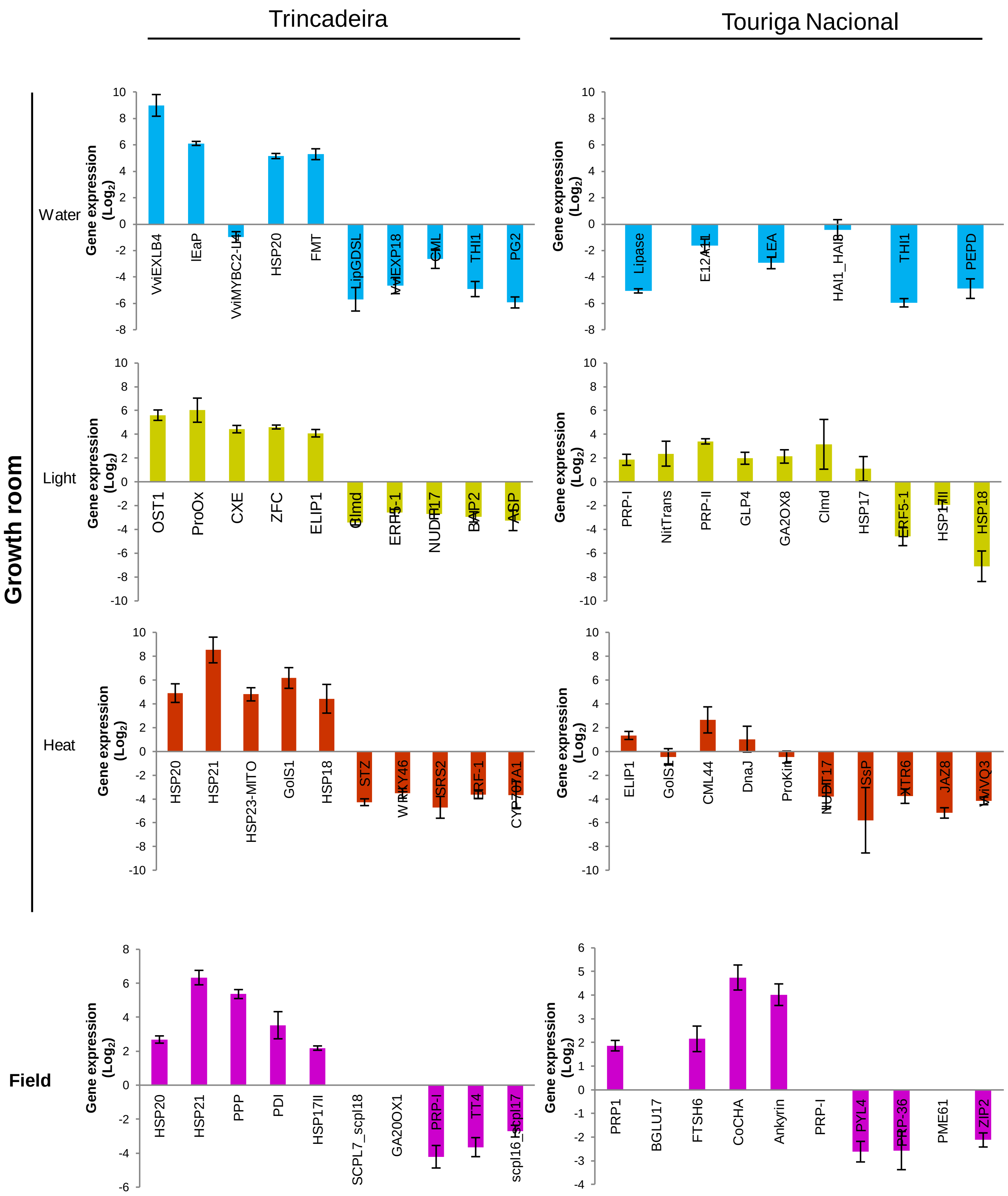

Additional file 8. Validation of the microarray gene expression by RT-qPCR. RT-qPCR was performed in Trincadeira and Touriga Nacional leaf samples from individual abiotic stresses. Gene expression for the five most up and down-regulated genes in the individual abiotic stress in both cultivars by RT-qPCR analysis. Genes more up or down regulated after water deficit (blue); after light stress (light green); after heat stress (red), after field experiments (purple). Gene annotation as in Tables 2 and 3; r, correlation coefficient.
