# Supplementary material for: Transcriptomic comparison between two Vitis vinifera L. varieties (Trincadeira and Touriga Nacional) in abiotic stress conditions
Source: BMC Plant Biol. 2016 Oct 12;16:224. doi: 10.1186/s12870-016-0911-4 (PMC5062933; doi:10.1186/s12870-016-0911-4)
Supplement: Additional file 9: — Correlation between the gene expression from the microarray and RT-qPCR analysis. (PDF 141 kb) [file 12870_2016_911_MOESM9_ESM.pdf]

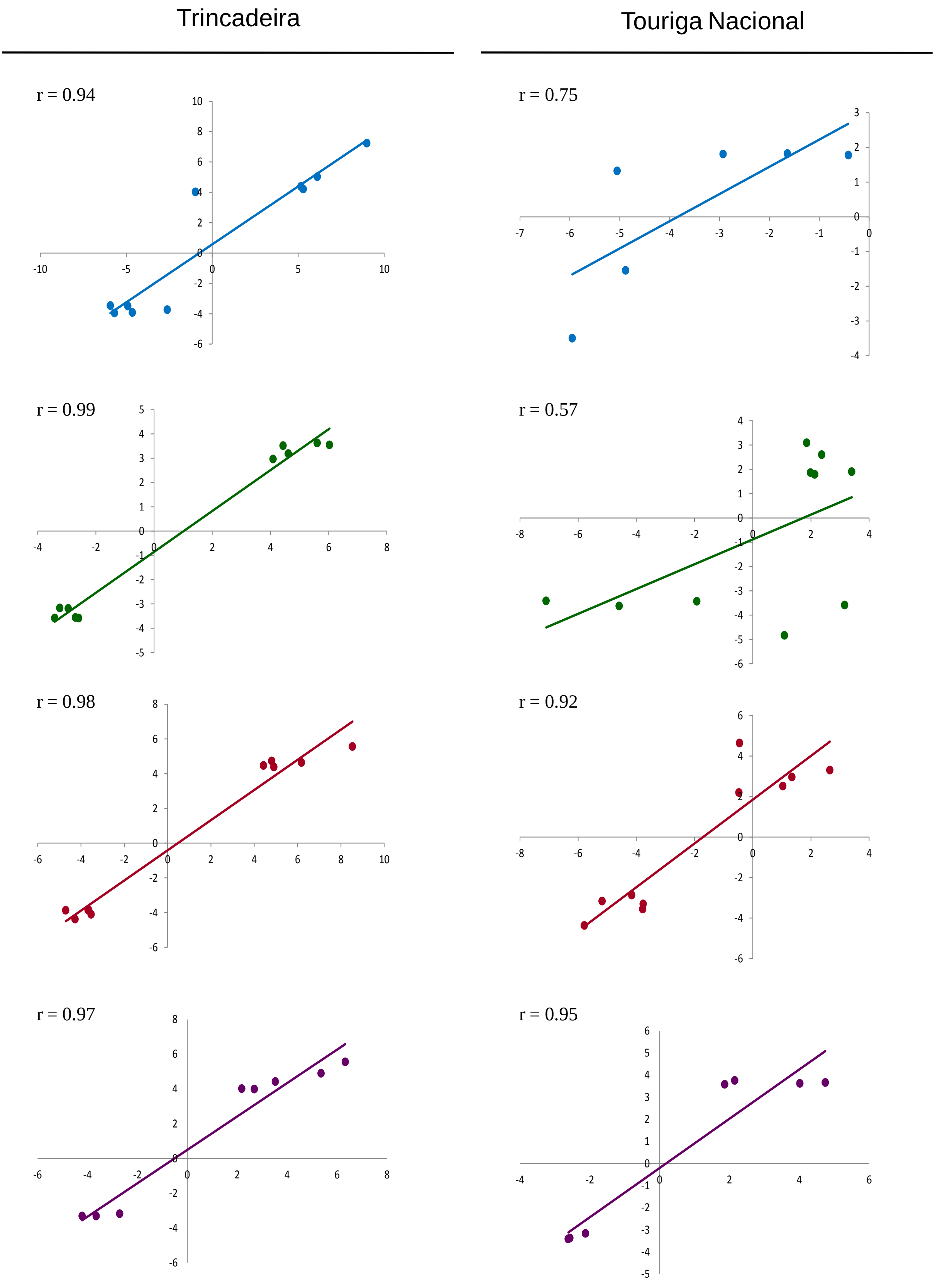

Additional file 9. Correlation between the gene expression from the microarray and RT-qPCR analysis. Correlation (**r**) between the gene expression from microarray and qPCR in $\log_2$ for the same genes in both cultivars Trincadeira (TR) and Touriga Nacional (TN). Water deficit (Blue); Light stress (green); Heat stress (red); Field (purple). X axis – expression values from qRT-PCR, Y axis – expression values from microarray analysis.
