# Supplementary material for: Transcriptomic comparison between two Vitis vinifera L. varieties (Trincadeira and Touriga Nacional) in abiotic stress conditions
Source: BMC Plant Biol. 2016 Oct 12;16:224. doi: 10.1186/s12870-016-0911-4 (PMC5062933; doi:10.1186/s12870-016-0911-4)
Supplement: Additional file 10: — Analyses of responsive transcripts in the Unknown Functional Category. (PDF 453 kb) [file 12870_2016_911_MOESM10_ESM.pdf]

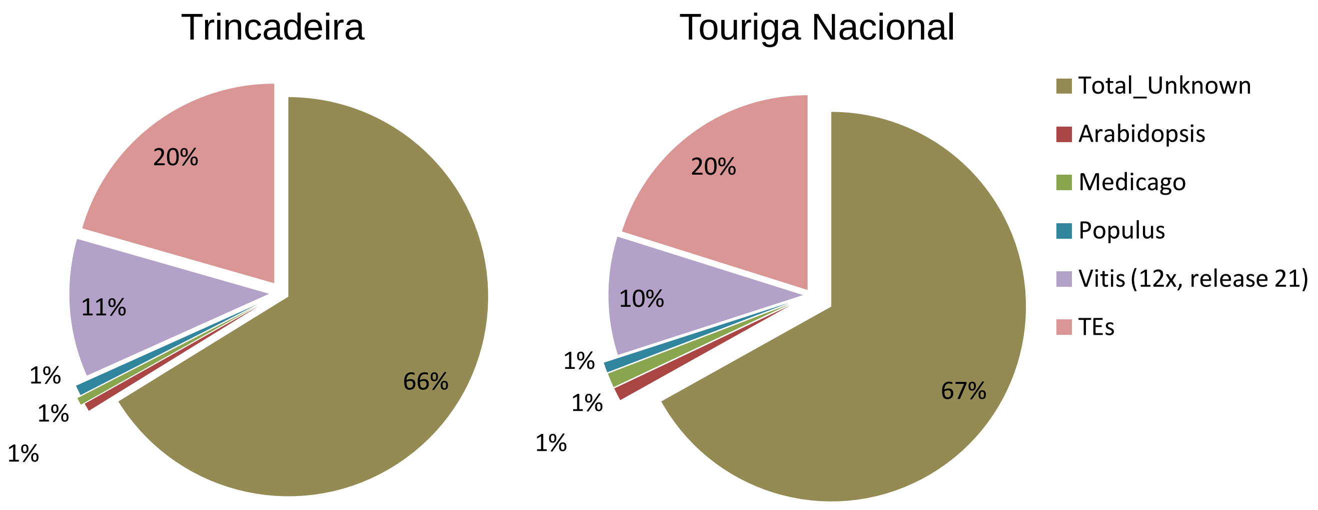

Figure S1. Genes with Unknown functional annotation but treatment responsive in Trincadeira and Touriga Nacional. TEs, transposable elements.

Table S1. Number of exclusive “Unknown” genes in each treatment matching sequences of a transposon database(http://www.girinst.org/).

	Nº Unknow	Nº matches	%		Nº Unknow	Nº matches	%
**TR W**	299	85	28	**TN W**	20	5	25
**TR L**	243	73	30	**TN L**	28	10	36
**TR H**	42	17	40	**TN H**	108	30	28
**Field**	160	44	27.5	**Field**	318	97	30.5

Table S2. Genes classified as “Unknown” in Trincadeira exclusive to each treatment submitted to a transposon database(http://www.girinst.org/)

	**TR W**		**TR L**		**TR H**		**Field**	
**Repeat Class**	Fragments	Length	Fragments	Length	Fragments	Length	Fragments	Length
Simple repeat	2	118	1	409	-	-	1	52
Transposable Element	95	6834	83	6340	17	1460	51	3227
DNA transposon	33	2296	32	2374	7	469	15	952
Endogenous Retrovirus	5	233	1	56	-	-	2	69
LTR Retrotransposon	45	3270	34	2233	6	660	20	1296
Non-LTR Retrotransposon	12	1035	16	1677	4	331	14	910
Pseudogene	1	317	8	516	2	74	-	-
**Total**	98	7269	92	7265	17	1534	52	3279

Table S3. Genes classified as “Unknown” in Touriga Nacional exclusive to each treatment submitted to a transposon database(http://www.girinst.org/)

	**TN W**		**TN L**		**TN H**		**Field**	
**Repeat Class**	Fragments	Length	Fragments	Length	Fragments	Length	Fragments	Length
Simple repeat	-	-	1	73	2	134	3	393
Transposable Element	5	243	8	692	37	2660	119	8495
DNA transposon	3	143	5	448	14	1084	44	3310
Endogenous Retrovirus	-	-	-	-	3	179	4	247
LTR Retrotransposon	1	43	1	43	15	970	51	3492
Non-LTR Retrotransposon	1	57	2	201	5	427	20	1446
Pseudogene	-	-	1	37	-	-	-	-
**Total**	5	243	10	765	39	2794	122	8888

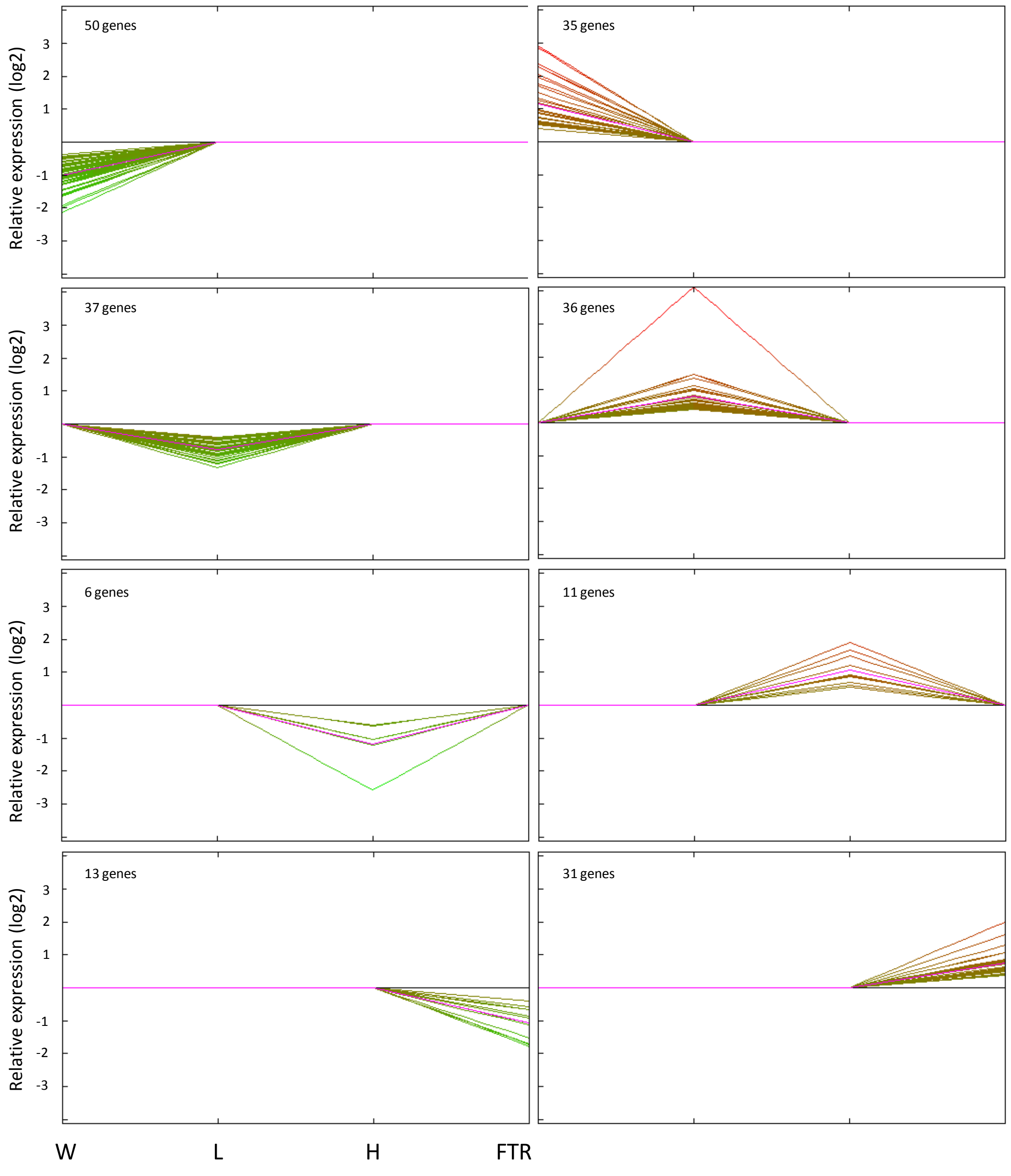

Figure S2. Exclusive TE expression profiles during stress treatments and in field trial in Trincadeira. In Trincadeira 219 genes gave homology with transposable elements when submitted to a TE database (http://www.girinst.org/). Those genes were grouped into 8 clusters using the k-means algorithm that gave the best representation of the different profiles. y-axis- relative gene expression value (in log_2 scale) for each gene; x-axis- type of stress, W, L, H and FTR for water, high light, heat stress and field samples.

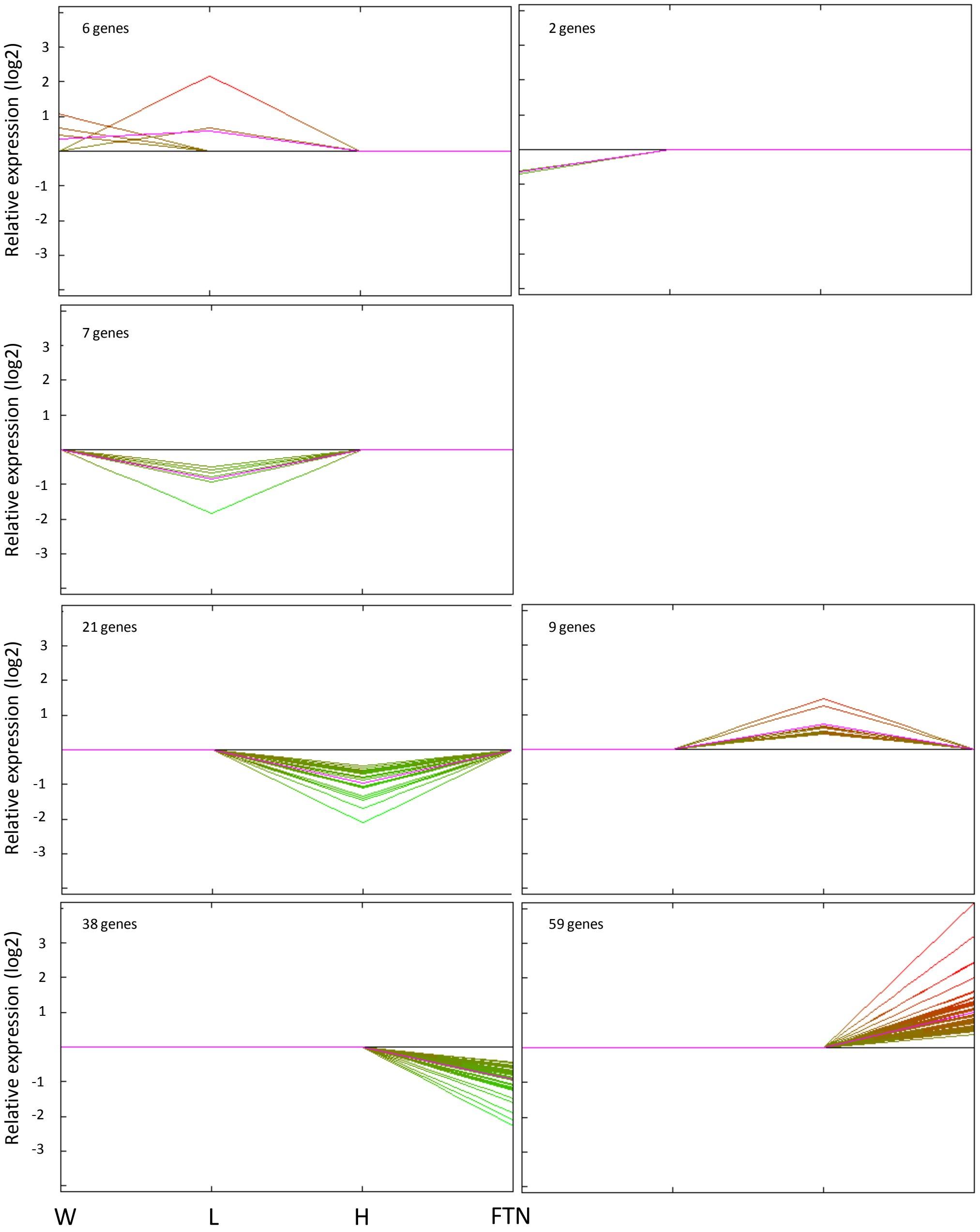

Figure S3. Exclusive TE expression profiles during stress treatments and in field trial in Touriga Nacional. In Touriga Nacional 142 genes gave homology with transposable elements when submitted to a TE database (http://www.girinst.org/). Those genes were grouped into 8 clusters using the k-means algorithm that gave the best representation of the different profiles. y-axis- relative gene expression value (in log_2 scale) for each gene; x-axis- type of stress, W, L, H and FTN for water, high light, heat stress and field samples.

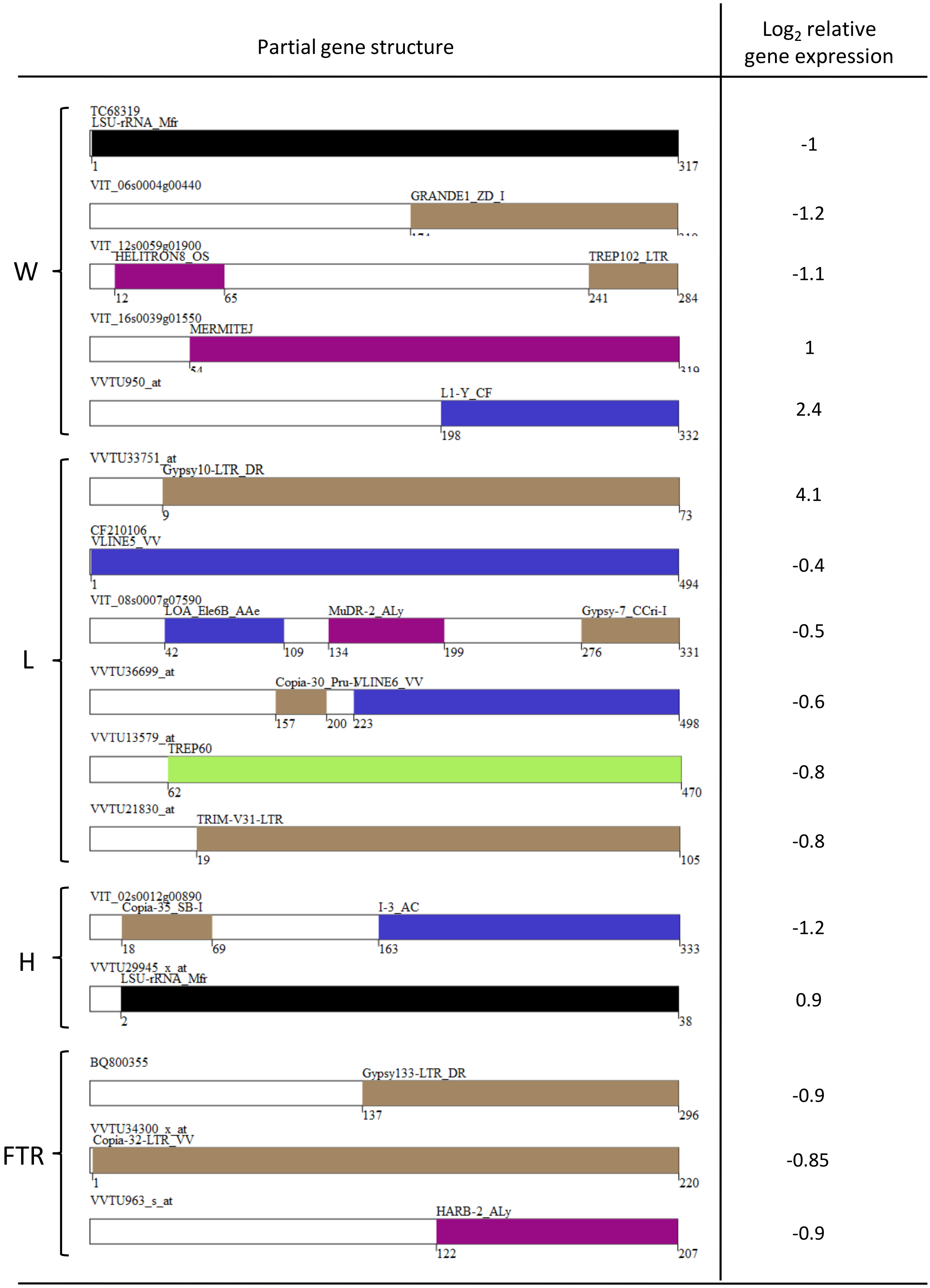

Figure S4. Examples of TE fragments expressed after stress treatment as well as in field samples, in Trincadeira. The expression values are shown on the right. TE database was: http://www.girinst.org/. W, water stress; L, high light stress; H, heat stress; FTR, field, Trincadeira samples.

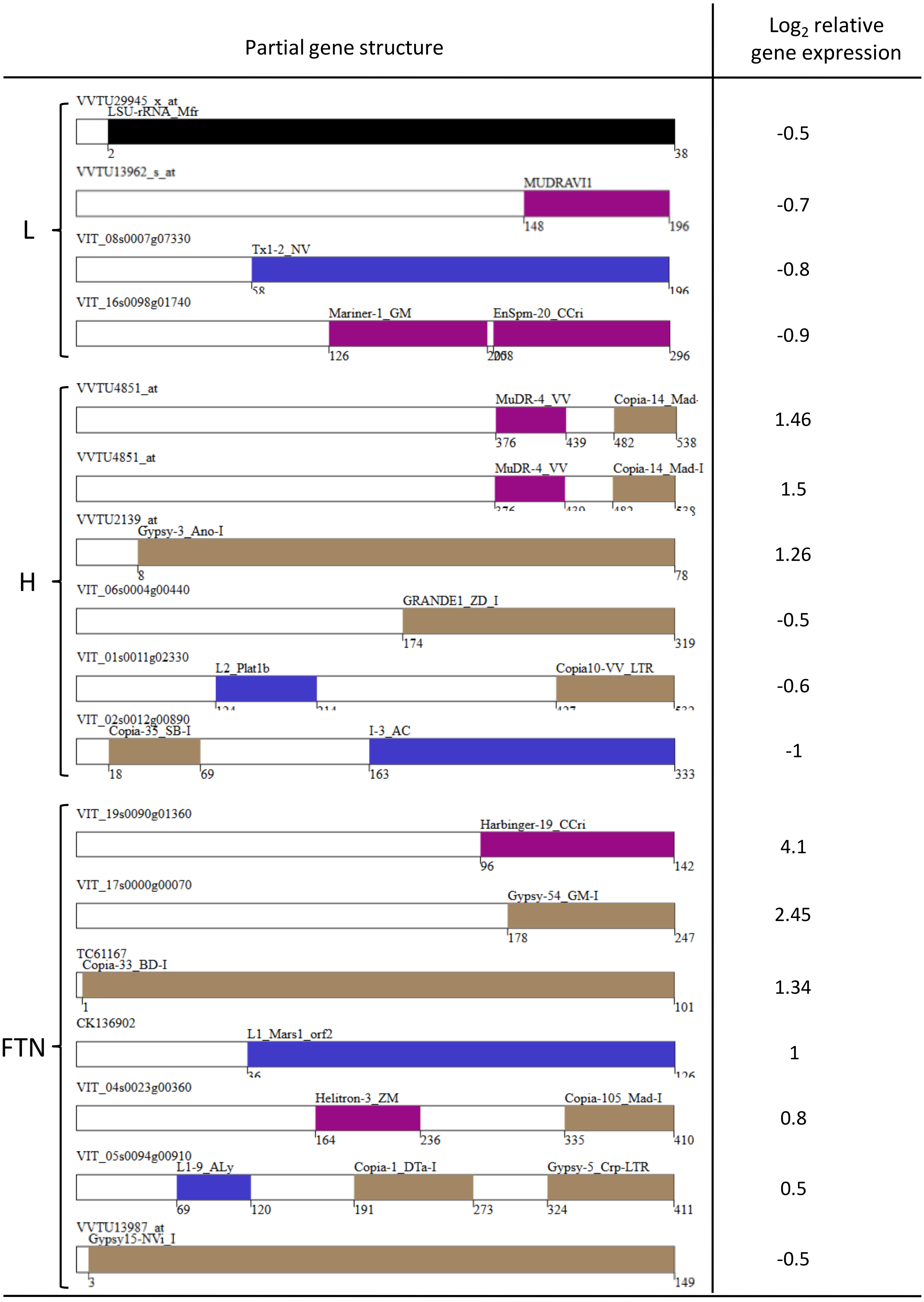

Figure S5. Examples of TE fragments expressed after stress treatments as well as in field samples, in Touriga Nacional. The expression values are shown on the right. TE database was: http://www.girinst.org/. W, water stress; L, light stress; H, heat stress; FTN, field, Touriga Nacional samples.

Partial gene structure	Log_2 relative gene expression

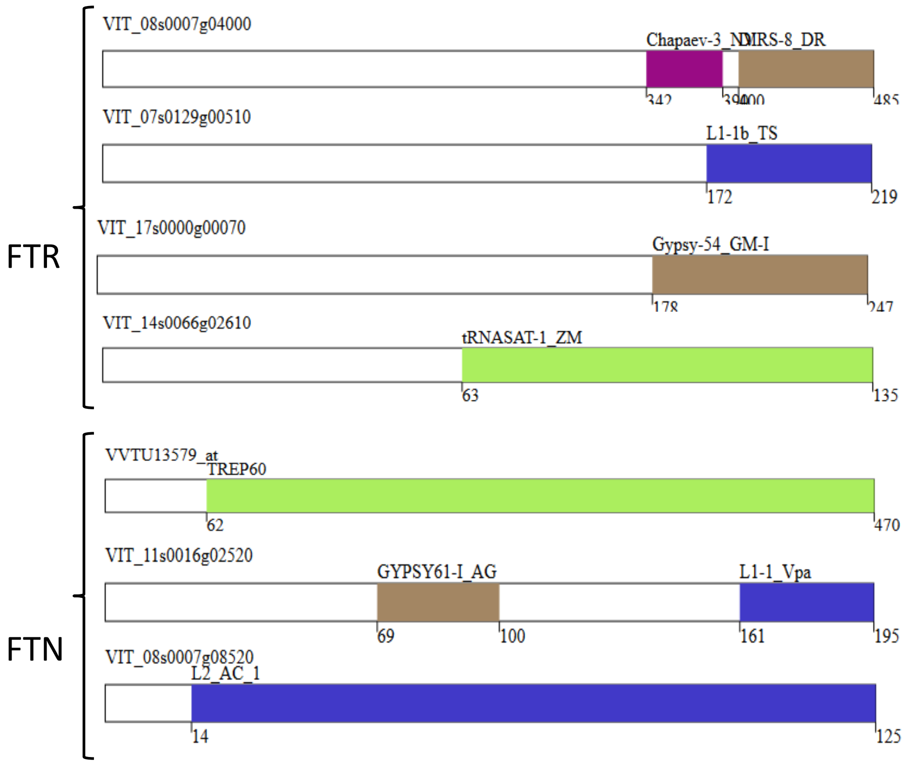

Sample	Gene	Water	Light	Heat	Field
FTR	VIT_08s0007g04000	2.9		3.5	5.2
FTR	VIT_07s0129g00510	3.9			0.68
FTR	VIT_17s0000g00070	3.2		3.4	3.5
FTR	VIT_14s0066g02610	-1.4	-2.2	-2.15	0.78
FTN	VVTU13579_at			-1.89	2.1
FTN	VIT_11s0016g02520	-1.93		3.14	1.83
FTN	VIT_08s0007g08520	-1.37			-1.73

Figure S6. Examples of TE fragments expressed in several stresses as analysed in field samples. The expression values are shown on the right. TE database was: http://www.girinst.org/. FTR and FTN, Trincadeira and Touriga Nacional field sample, respectively.
